# Excitation–inhibition imbalance links amyloid pathophysiology to cognition in non‐demented individuals

**DOI:** 10.1002/alz.71891

**Published:** 2026-09-30

**Authors:** Shaoshi Zhang, Sebastian N. Roemer‐Cassiano, Joyce Ruifen Chong, Tian Fang, Yan Li, Ziwen Wang, Tianchu Zeng, Xin Li, Gustavo Deco, Narayanaswamy Venketasubramanian, Juan Helen Zhou, Christopher Li‐Hsian Chen, Alzheimer's Disease Neuroimaging Initiative, Mitchell K. P. Lai, Nicolai Franzmeier, B. T. Thomas Yeo

**Affiliations:** ^1^ Centre for Sleep & Cognition & Centre for Translational Magnetic Resonance Research Yong Loo Lin School of Medicine National University of Singapore Singapore Singapore; ^2^ Department of Medicine Healthy Longevity Translational Research Programme Human Potential Translational Research Programme & Institute for Digital Medicine (WisDM) Yong Loo Lin School of Medicine National University of Singapore Singapore Singapore; ^3^ N.1 Institute for Health & Institute for Digital Medicine National University of Singapore Singapore Singapore; ^4^ Department of Electrical and Computer Engineering National University of Singapore Singapore Singapore; ^5^ Integrative Sciences and Engineering Programme (ISEP) National University of Singapore Singapore Singapore; ^6^ Institute for Stroke and Dementia Research LMU Munich Munich Germany; ^7^ Department of Neurology LMU Hospital LMU Munich Munich Germany; ^8^ Wisconsin Alzheimer's Disease Research Center School of Medicine and Public Health University of Wisconsin–Madison Madison Wisconsin USA; ^9^ Department of Pharmacology, Yong Loo Lin School of Medicine National University of Singapore Singapore Singapore; ^10^ Memory, Ageing and Cognition Centre Departments of Pharmacology and Psychological Medicine Yong Loo Lin School of Medicine National University of Singapore Singapore Singapore; ^11^ Aging Research Center Karolinska Institute and Stockholm University Stockholm Sweden; ^12^ Center for Brain and Cognition Department of Technology and Information Universitat Pompeu Fabra Barcelona Spain; ^13^ Institució Catalana de la Recerca i Estudis Avançats Universitat Barcelona Barcelona Spain; ^14^ Raffles Neuroscience Centre, Raffles Hospital Singapore Singapore; ^15^ Munich Cluster for Systems Neurology (SyNergy) Munich Germany; ^16^ Department of Psychiatry and Neurochemistry Institute of Neuroscience and Physiology University of Gothenburg, The Sahlgrenska Academy Gothenburg Sweden; ^17^ Athinoula A. Martinos Center for Biomedical Imaging Massachusetts General Hospital Charlestown Massachusetts USA

**Keywords:** Alzheimer's disease, amyloid beta, amyloid pathophysiology, biomarkers, biophysical modeling, cognition, plasma phosphorylated tau

## Abstract

**INTRODUCTION:**

Excitation–inhibition (E/I) imbalance has been proposed as an early circuit‐level mechanism in Alzheimer's disease (AD), but its relationship to molecular pathology and cognition in humans remains unclear.

**METHODS:**

We integrated biophysical modeling of resting‐state functional magnetic resonance imaging with cerebrospinal fluid (CSF) and plasma biomarkers to examine whether E/I ratio links amyloid pathophysiology to memory in non‐demented individuals across two cohorts from North America (*N* = 302; CSF amyloid beta 42 [Aβ42]) and Singapore (*N* = 240; plasma phosphorylated tau 217 [p‐tau217]).

**RESULTS:**

Elevated E/I ratio was associated with lower CSF Aβ42 and higher plasma p‐tau217, with greater sensitivity in sensory–motor regions. In contrast, elevated E/I ratio in association cortices was more strongly related to worse memory function. Mediation analyses indicated E/I ratio partially accounted for the AD biomarker–memory relationship.

**DISCUSSION:**

E/I imbalance represents an intermediate, systems‐level circuit mechanism that links AD pathophysiology to cognitive impairment, offering novel insight into early neurophysiological changes along the AD continuum.

## Highlights

- A putative marker of cortical excitation–inhibition (E/I) ratio was estimated in vivo from resting‐state functional magnetic resonance imaging using a biophysically plausible neural mass model in two independent datasets of non‐demented individuals.
- Greater amyloid pathophysiology, indexed by lower cerebrospinal fluid amyloid beta 42 and higher plasma phosphorylated tau 217, was associated with elevated cortical E/I ratio.
- The association between E/I ratio and amyloid pathophysiology was strongest in sensory–motor regions, whereas the association between E/I ratio and memory performance was strongest in higher order association cortices, particularly the default network.
- E/I ratio partially mediated the relationship between Alzheimer's disease biomarkers and memory performance, identifying it as a systems‐level circuit mechanism linking amyloid pathophysiology to cognition.

## 1 BACKGROUND

Alzheimer's disease (AD) is defined as a pathophysiological continuum in which amyloid beta (Aβ) promotes tau aggregation, thereby driving neurodegeneration and cognitive decline.^1^, ^2^, ^3^, ^4^ Substantial AD‐related pathology can already be detected in cognitively normal (CN) individuals and those with mild cognitive impairment (MCI), highlighting the importance of early intervention to slow or prevent disease progression.^5^, ^6^, ^7^ However, the mechanisms through which early amyloid pathology translates into cognitive impairment remain largely unresolved.

Early disruption of the excitation–inhibition (E/I) balance is thought to be a circuit‐level mechanism linking AD pathology and cognitive decline.^8^, ^9^, ^10^ As amyloid pathology progresses, soluble Aβ aggregates into oligomeric and fibrillar forms, with oligomers particularly implicated in synaptic dysfunction.^11^ Aβ oligomers have been shown to enhance excitatory transmission, weaken inhibitory control, and alter intrinsic neuronal excitability, thereby biasing cortical circuits toward excitation‐dominant dynamics.^12^, ^13^ The resulting increase in E/I ratio can destabilize local circuit dynamics and impair coordinated network activity, ultimately contributing to network fragility and decreasing structural integrity along the AD continuum.^14^, ^15^, ^16^, ^17^

Human functional magnetic resonance imaging (fMRI) studies have reported hippocampal hyperactivation in CN and MCI individuals during cognitive tasks, often interpreted as a compensatory response.^18^, ^19^, ^20^ However, these studies rarely incorporate direct measures of AD pathology, limiting their ability to link altered neural excitability to underlying molecular processes.^21^ Mechanistic insights from animal models and computational modeling in human imaging data have shown how amyloid drives hyperexcitability, but these studies typically do not directly assess cognitive outcomes.^22^, ^23^, ^24^, ^25^

The development of cerebrospinal fluid (CSF) and plasma biomarkers has made it possible to quantify AD pathophysiology in vivo. While CSF biomarkers provide a direct and well‐validated measure of soluble amyloid and tau,^26^, ^27^, ^28^ their use is limited by the need for lumbar punctures. Recent advances in ultrasensitive plasma biomarkers, in contrast, offer a minimally invasive and more scalable alternative, enabling investigation of AD‐related molecular changes in larger cohorts.^29^, ^30^, ^31^ Importantly, these fluid biomarkers closely reflect ongoing amyloid‐related molecular pathophysiology in early AD,^32^ whereas positron emission tomography (PET) primarily detects fibrillar deposition that has accumulated over years.^33^ Although PET studies have linked amyloid burden to E/I imbalance,^17^, ^34^, ^35^ it remains unclear whether more dynamic molecular measures captured by CSF or plasma biomarkers relate to E/I ratio and, in turn, to cognitive performance in humans.

In this study, we integrate biophysical modeling, multimodal AD biomarkers, and cognitive assessments to investigate whether E/I ratio mediates the relationship between AD pathophysiology and cognition in two independent samples of CN and MCI individuals from North America and Singapore. We have previously used a biophysical model to derive a putative in vivo marker of the E/I ratio from resting‐state fMRI, which is sensitive to pharmacological modulation of GABAergic inhibition.^36^ Here, we test the associations of the E/I ratio marker with CSF Aβ42 and plasma phosphorylated tau 217 (p‐tau217), and perform mediation analyses to assess whether the E/I ratio links molecular pathology to cognitive decline. Converging evidence across biomarkers reveals cortex‐wide increases in the E/I ratio, with heightened sensitivity to amyloid in sensory–motor regions and stronger cognitive associations in the default network, providing novel systems‐level insight into early AD circuit mechanisms.

**RESEARCH IN CONTEXT**

1. **Systematic review**: Disruption of the cortical excitation–inhibition (E/I) balance has been proposed as an early circuit‐level mechanism linking amyloid beta (Aβ) pathology to cognitive decline in Alzheimer's disease (AD), with converging support from animal models and task‐based human functional magnetic resonance imaging (fMRI) studies. However, prior human studies have rarely combined in vivo estimates of E/I balance with molecular biomarkers of AD pathophysiology, and the role of E/I imbalance as a mediator between amyloid burden and cognition remains unclear.
2. **Interpretation**: Using biophysical modeling of resting‐state fMRI in two independent cohorts of non‐demented individuals from North America (cerebrospinal fluid [CSF] Aβ42) and Singapore (plasma phosphorylated tau 217), we found that greater amyloid pathophysiology was associated with elevated cortical E/I ratio. The amyloid–E/I association was most pronounced in sensory–motor regions, whereas elevated E/I ratio in higher order association cortices—particularly the default network—was most strongly related to worse memory performance. Mediation analyses indicated that E/I ratio partially accounts for the relationship between AD biomarkers and memory performance, supporting E/I imbalance as a systems‐level circuit mechanism linking molecular pathology to cognition in humans.
3. **Future directions**: Longitudinal studies combining fMRI‐based E/I estimation with CSF and plasma biomarker measurements are needed to characterize how circuit dynamics evolve over time and whether E/I imbalance predicts subsequent cognitive decline. Extending the current framework to individuals with more advanced disease stages, and testing whether pharmacological or non‐invasive modulation of cortical excitability slows cognitive decline, will be important next steps toward translating E/I balance into a therapeutic target.

## 2 METHODS

### 2.1 Alzheimer's Disease Neuroimaging Initiative dataset

For this study, we included CN and participants with MCI from three phases of the Alzheimer's Disease Neuroimaging Initiative (ADNI) dataset: ADNI‐GO, ADNI‐2, and ADNI‐3. CN participants met the following criteria: Mini‐Mental State Examination (MMSE) score > 24, Clinical Dementia Rating (CDR) of 0, and absence of depression. MCI participants were required to have an MMSE score > 24, a CDR of 0.5, objective memory impairment on the education‐adjusted Wechsler Memory Scale II, and preserved activities of daily living.

We started from an initial sample of 970 participants. Participants were excluded if the interval between resting‐state fMRI acquisition and CSF collection exceeded 1 year, or if the interval between fMRI acquisition and clinical diagnosis exceeded 1 year. Application of these criteria resulted in a final sample of 302 participants, whose demographic characteristics are summarized in Table S1 in supporting information. Resting‐state fMRI acquisition protocols varied across ADNI phases, and a summary of imaging parameters is provided in Table S2 in supporting information. CSF samples were collected in site‐provided tubes and transferred to polypropylene tubes. Within 1 hour of collection, samples were frozen on dry ice and shipped overnight on dry ice to the ADNI Biomarker Core Laboratory at the University of Pennsylvania Medical Center. After thawing at room temperature for 1 hour with gentle mixing, 0.5 mL aliquots were prepared and stored in barcoded polypropylene vials at −80°C. CSF Aβ40 and Aβ42 concentrations were subsequently measured using the Roche Elecsys immunoassay platform.

### 2.2 ADNI fMRI preprocessing

Resting‐state fMRI data from each ADNI participant were slice‐time corrected, motion corrected, and aligned with the structural image using boundary‐based registration.^37^ Six head motion parameters together with their temporal derivatives, and the top five aCompCor components, were regressed from the functional data, which were then projected onto the FreeSurfer fsaverage6 surface.

The projected functional time courses were averaged within each Desikan–Killiany cortical region,^38^ yielding a 68 × T matrix per participant, where T is the number of fMRI frames. Static functional connectivity (FC) matrices (68 × 68) were computed by correlating the regional time courses. Functional connectivity dynamics (FCD) matrices were computed using sliding‐window correlations of FC matrices with a 60‐second window;^39^, ^40^ full computational details are provided in Methods S1 in supporting information.

### 2.3 Memory, Ageing and Cognition Centre Harmonization dataset

Memory, Ageing and Cognition Centre (MACC) Harmonization data collection procedures and sample characteristics have been previously described in detail.^41^ We started from an initial sample of 575 participants. For this study, we only considered CN and cognitively impaired but not demented (CIND) participants. Diagnostic criteria have been reported previously.^42^ Briefly, CN participants showed no objective cognitive impairment on a locally validated neuropsychological test battery.^43^ CIND was defined as impairment in at least one cognitive domain on neuropsychological testing without meeting Diagnostic and Statistical Manual of Mental Disorders 4th Edition criteria for dementia. We will refer to CIND participants as MCI participants to be consistent with the ADNI terminology.

Participants were excluded if the interval between fMRI acquisition and blood collection exceeded 1 year, or if the interval between fMRI acquisition and clinical diagnosis exceeded 1 year. These criteria yielded a final sample of 240 participants, whose demographic characteristics are summarized in Table S3 in supporting information. All fMRI data were collected on the same Siemens Tim Trio 3T scanner. The scanning protocol and acquisition parameters have been previously described in detail.^44^ Briefly, fMRI was acquired using the following parameters: repetition time = 2300 ms; echo time = 25 ms; flip angle = 90°; field of view = 192 × 192 mm^2^; effective voxel resolution = 3.0 × 3.0 × 3.0 mm^3^; 124 volumes. As for the plasma biomarker measurements, non‐fasting blood was collected into ethylenediaminetetraacetic acid (EDTA)‐containing tubes and centrifuged at 2000 g for 10 minutes at 4°C. Plasma was extracted, mixed well, aliquoted, and stored at −80°C until use. All biomarkers were measured on the single molecule array (Simoa) HD‐1 or HD‐X platforms (Quanterix Corp.). Plasma p‐tau217 and p‐tau181 were measured using the commercial ALZpath p‐Tau 217 Advantage PLUS assay (item 104570) and Simoa pTau‐181 Advantage V2 assay (item 103714), respectively.

### 2.4 MACC fMRI preprocessing

Resting‐state fMRI data were preprocessed with the following steps. The first four frames of the fMRI data were removed. The remaining functional data were then slice time‐corrected, motion‐corrected, and aligned with structural image using boundary‐based registration.^37^ Six head motion parameters together with their temporal derivatives, and the top five aCompCor components were regressed from the functional data. Last, the functional data were then projected onto FreeSurfer fsaverage6 surface space. FC and FCD of the MACC dataset were computed in the same manner as the ADNI dataset.

### 2.5 Parametric feedback inhibition control model

The parametric feedback inhibition control (pFIC) model is a neural mass model derived from a principled mean‐field reduction of spiking neuronal network models,^15^, ^45^, ^46^ providing a biophysically plausible formulation of circuit‐level dynamics. Briefly, each cortical region is described using coupled ordinary differential equations that capture the activity of local excitatory and inhibitory neuronal populations^15^, ^45^, ^46^ (Figure 1). Long‐range connectivity between excitatory populations is provided by the structural connectome, yielding a large‐scale model of cortical dynamics (Figure 1). Compared to the original feedback inhibition control (FIC) model,^15^ pFIC parameterizes the local excitatory‐to‐excitatory and excitatory‐to‐inhibitory connection strengths and the noise amplitude as a linear combination of the T1w/T2w ratio map and the principal functional connectivity gradient,^36^, ^47^ reducing the number of free parameters to 10. The full system of equations and parameter settings is provided in the Methods S2 in supporting information.

**FIGURE 1:**
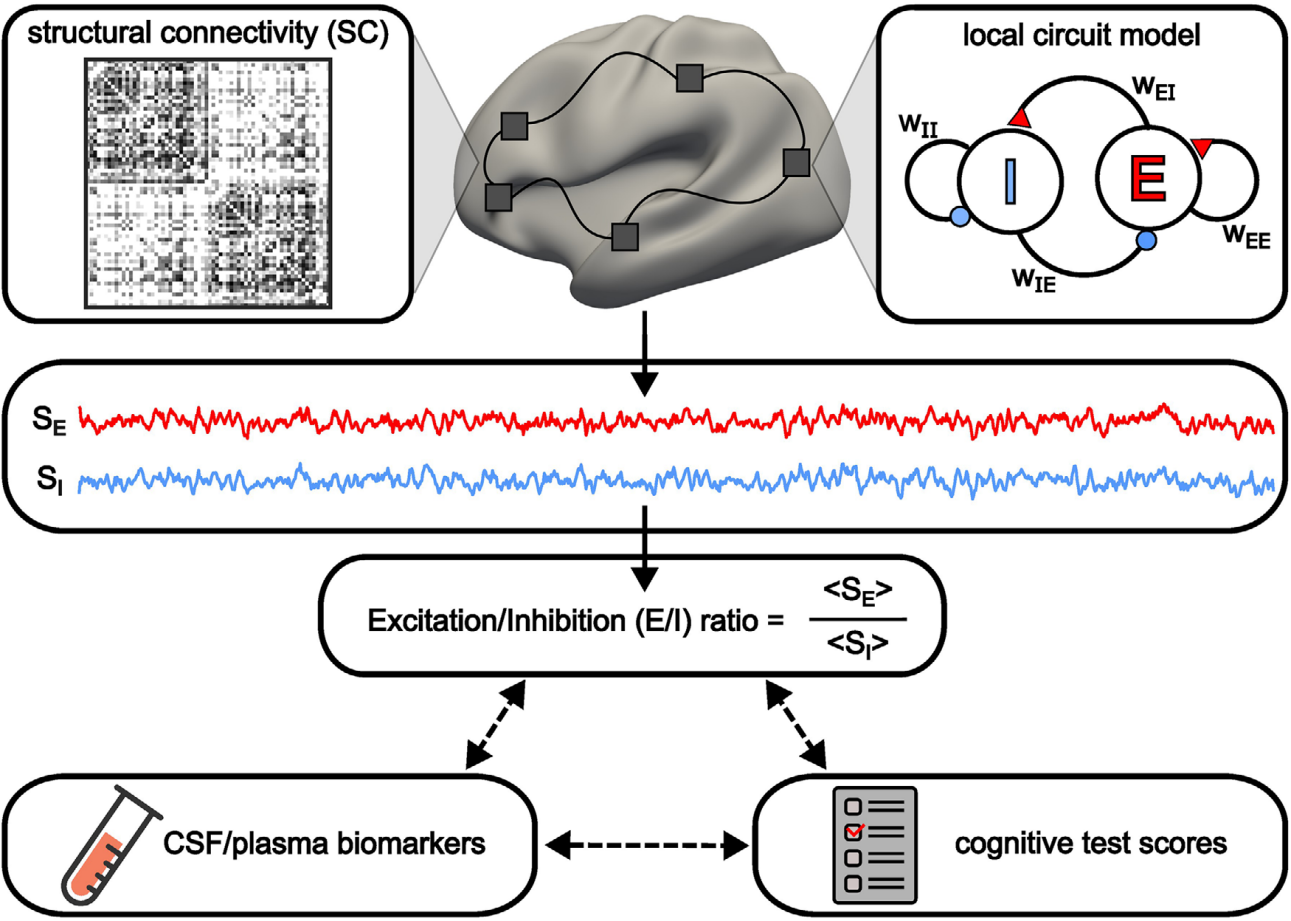
Analysis overview. Individual‐level markers of excitation–inhibition (E/I) ratio were estimated using our previously established parametric feedback inhibition control (pFIC) model.^36^ The pFIC model is a neural mass model, derived from principled mean field reduction of spiking neuronal models.^15^, ^45^, ^46^ Briefly, the pFIC model describes local circuits as coupled excitatory (“E”) and inhibitory (“I”) neuronal populations governed by differential equations. Excitatory and inhibitory connections are indicated by red triangles and blue circles, respectively, with connection strengths denoted by *wxy* (from population *x* to *y*). Structural connectivity (SC) links excitatory populations between cortical regions. The pFIC model parameters were estimated by optimizing fit to resting‐state functional magnetic resonance imaging. Simulated synaptic gating variables (SE and SI) were then used to derive the E/I ratio as the ratio of their temporal averages.^36^ Cerebrospinal fluid (CSF) and plasma biomarkers were obtained from the respective datasets, including CSF amyloid beta 42 from the Alzheimer's Disease Neuroimaging Initiative^93^ and plasma phosphorylated tau 217 from the Memory, Ageing and Cognition Centre Harmonization dataset.^94^, ^95^ Mediation analyses were then conducted to examine relationships among CSF/plasma biomarkers (independent variables), E/I ratio (mediator), and cognitive test performance (dependent variables)

Simulated excitatory and inhibitory synaptic gating variables (*S_E_* and *S_I_*) were obtained for each cortical region, and simulated fMRI blood‐oxygenation‐level–dependent signals were generated by passing *S_E_* through the Balloon–Windkessel hemodynamic model.^15^, ^48^, ^49^ Motivated by previous rodent studies,^50^ the regional E/I ratio was defined as the ratio of the temporal averages of *S_E_* and *S_I_* (Figure 1), and the mean cortical E/I ratio was the average across all cortical regions. The resulting marker has been validated against pharmacological manipulation of GABAergic inhibition with alprazolam and is spatially aligned with PET measurements of benzodiazepine receptor density.^36^ Here, we examined the relationships of the putative E/I ratio marker with CSF/plasma biomarkers and cognitive performance (Figure 1).

### 2.6 Individual‐level pFIC optimization and E/I ratio estimation

The 10 unknown parameters of the pFIC model were optimized by maximizing fit to empirical static FC and FCD.^36^ For the optimization algorithm, we used the covariance matrix adaptation evolution strategy (CMA‐ES).^47^, ^51^ The parameter set with the lowest cost was retained, and individual‐level E/I ratio estimates were obtained by averaging across 1000 simulations with independent noise instantiations. As the fMRI data were collected from multiple scanning sites in the ADNI dataset, the estimated E/I ratios were harmonized for each region of interest independently using ComBat across participants.^52^ No additional site harmonization was required for the MACC dataset as the data were collected with a single scanner. Full procedural details are provided in Methods S3 in supporting information.

Although the E/I ratio was estimated separately for each participant, subsequent analyses estimated associations across participants rather than within individuals. Accordingly, the results support inferences at the cohort and regional levels, but do not establish individual‐level relationships or support individual‐level prediction or clinical interpretation.

### 2.7 General linear modeling of associations between mean E/I ratio and CSF or plasma biomarkers

General linear modeling (GLM) was used to assess associations between the mean E/I ratio and CSF or plasma biomarkers across participants. Specifically, the mean E/I ratio was set as the dependent variable. In the ADNI dataset, CSF Aβ42 was included as the primary predictor and was log transformed prior to analysis. In the MACC dataset, log‐transformed plasma p‐tau217 was included as the primary predictor. Covariates included age, sex (female = 0, male = 1), head motion quantified by mean framewise displacement (FD), and years of education. All continuous variables were *z* scored across participants before model fitting. GLM coefficients were estimated using ordinary least squares. Statistical significance of regression coefficients was assessed using two‐sided *t* tests, with corresponding *P* values reported.

To assess the robustness of the main findings, we performed three sets of control analyses using the same GLM framework. First, we examined alternative biomarkers by replacing CSF Aβ42 with the CSF Aβ42/40 ratio, and plasma p‐tau217 with plasma p‐tau181, while keeping the covariate set unchanged. Second, we evaluated the impact of additional potential confounders by refitting the GLM with an expanded set of covariates, including body mass index (BMI), hypertension, diabetes, and smoking history, in addition to age, sex, head motion, and years of education. Third, to assess whether harmonization influenced the ADNI results, the analysis was repeated using E/I ratio estimates that were not harmonized across sites using ComBat.

### 2.8 Regional associations between E/I ratio and CSF or plasma biomarkers

To examine regional associations between cortical E/I ratio and AD biomarkers, we applied a GLM framework consistent with the mean E/I ratio analyses. Specifically, for each cortical region, we fitted a GLM with regional E/I ratio as the dependent variable and the biomarker (log‐transformed CSF Aβ42 in the ADNI dataset or log‐transformed plasma p‐tau217 in the MACC dataset) as the independent variable. In the main analyses, covariates included age, sex, head motion, and years of education. All continuous variables were *z* scored prior to model fitting.

Each cortical region was analyzed independently, yielding a regional standardized regression coefficient (β) that quantified the association between amyloid pathology and regional E/I ratio while controlling for covariates. The resulting β coefficients were visualized on the cortical surface and corrected for multiple comparisons across all 68 cortical regions (false discovery rate [FDR] *q* < 0.05). To characterize large‐scale network organization underlying regional heterogeneity, regional β coefficients were further correlated with the sensorimotor–association (SA) axis.^53^

### 2.9 Mediation analysis

We investigated whether E/I ratio mediates the association between CSF or plasma biomarkers and cognitive performance. In the ADNI dataset, the dependent variable was the Alzheimer's Disease Sequencing Project—Phenotype Harmonization Consortium (ADSP PHC) memory score.^54^ In the MACC dataset, a memory score was constructed from selected MMSE subscores and verbal memory tests to provide an analogous measure of memory performance based on comparable cognitive domains. Individual test scores were first *z* scored across participants, after which a latent memory factor was estimated using factor analysis. The resulting memory factor scores were used in the mediation analyses. The specific cognitive tests contributing to the MACC memory score and their factor loadings are detailed in Table S4 in supporting information.

For each dataset, mediation analyses were conducted using a GLM framework. The independent variable was the biomarker of interest (log‐transformed CSF Aβ42 in ADNI dataset or log‐transformed plasma p‐tau217 in MACC dataset), the mediator was the mean E/I ratio, and the dependent variable was the memory score. Age, sex, FD, and education were included as covariates in all pathways. All continuous variables were *z* scored prior to analysis. Path coefficients were estimated as follows:

- *a*: effect of the biomarker on E/I ratio, controlling for covariates.
- *b*: effect of E/I ratio on cognition, controlling for the biomarker and covariates.
- *c′*: direct effect of the biomarker on cognition, controlling for E/I ratio and covariates.
- *a × b*: indirect effect of the biomarker on cognition through the E/I ratio.

The indirect effect quantifies the extent to which E/I ratio mediates the association between amyloid burden and memory performance while controlling for demographic and motion‐related confounds.

We further performed mediation analyses at the regional level to assess the spatial heterogeneity in various path coefficients. For each cortical region, regional E/I ratio was used as the mediator between the biomarker of interest and the memory score. Regional estimates of the indirect effect (*a × b*) and the mediator‐outcome path (*b*) were visualized on the cortical surface and corrected for multiple comparisons across all 68 cortical regions (FDR *q* < 0.05).

The two‐sided *P* values of all path coefficients were assessed using 10,000 bootstrap samples.

### 2.10 Data and code availability

The ADNI data are publicly available (https://ida.loni.usc.edu/). The MACC dataset can be obtained via a data transfer agreement (https://macc‐sg.notion.site/Data‐Catalogue‐122e6ba0007d80688d11fa10b6fcbd01). Code for this study can be found here: https://github.com/ThomasYeoLab/CBIG/tree/master/stable_projects/fMRI_dynamics/Zhang2026_EIAD. Co‐authors (Z.W. and F.T.) reviewed the code before merging into the GitHub repository to reduce the chance of coding errors.

## 3 RESULTS

### 3.1 CSF Aβ42 is negatively associated with E/I ratio

We first examined the relationship between E/I ratio and CSF Aβ42. We hypothesized that lower CSF Aβ42 concentrations are linked to a higher E/I ratio. Analyses were conducted in 302 non‐demented participants (CN and MCI) from the ADNI dataset. Demographic characteristics of the sample are shown in Figure 2A. Consistent with our hypothesis, CSF Aβ42 showed a significant negative association with mean E/I ratio (Figure 2B,C), indicating that greater amyloid pathophysiology (i.e., lower CSF Aβ42) was associated with higher E/I ratio. Age was also positively associated with E/I ratio, suggesting a shift toward increased E/I ratio during aging (Figure 2B,D). Interestingly, the E/I ratio was more strongly associated with CSF Aβ42 than age.

**FIGURE 2:**
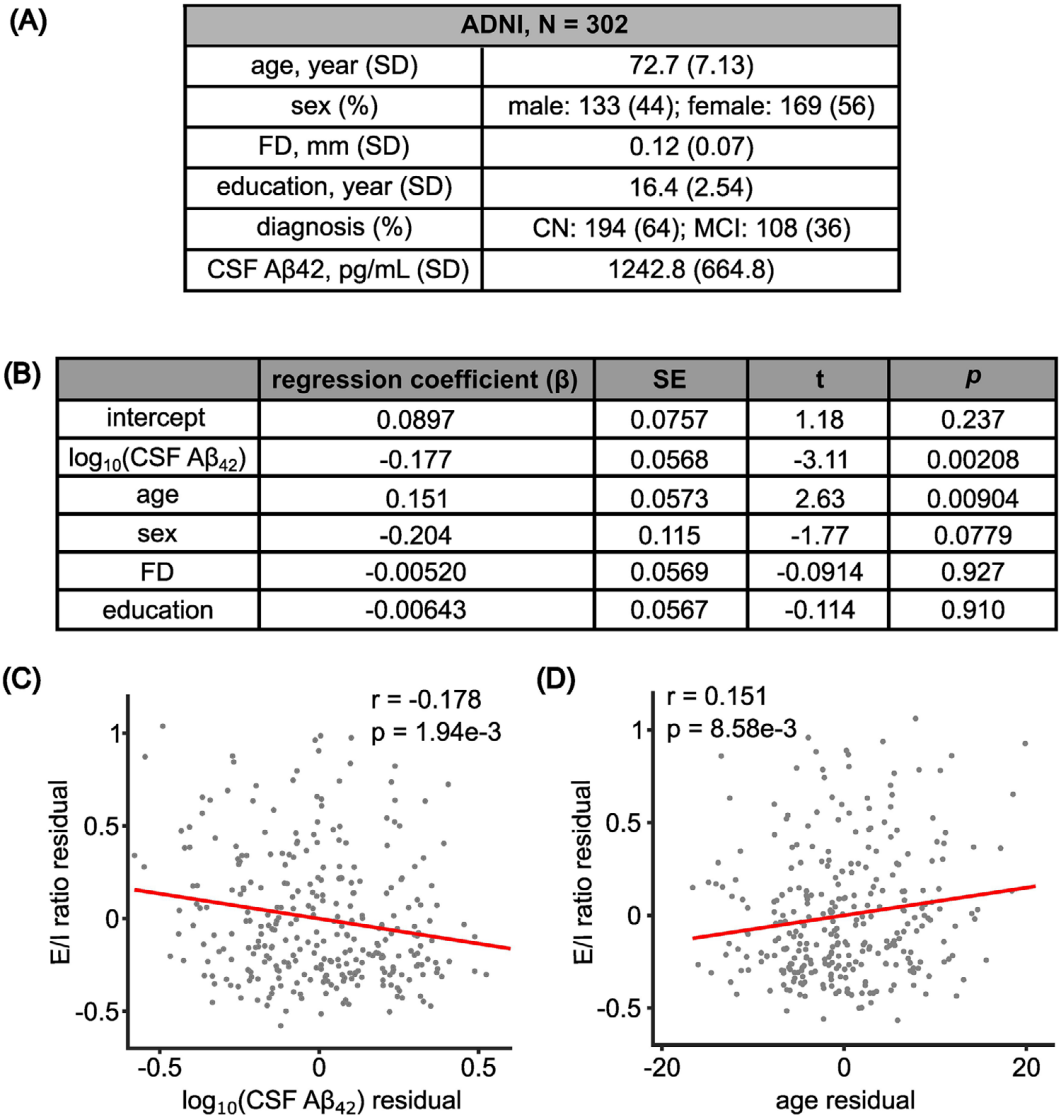
Association between mean cortical E/I ratio and CSF Aβ42. A, Participant characteristics in the ADNI dataset. B, GLM between mean cortical E/I ratio and log‐transformed CSF Aβ42, adjusting for age, sex (female = 0, male = 1), head motion (i.e., FD), and years of education. An intercept term is included. All continuous variables were *z* scored prior to model fitting. C, Visualization of the GLM result by showing a scatter plot of E/I ratio residuals (after regressing out covariates) with respect to log‐transformed CSF Aβ42 residuals (after regressing out covariates). Here, covariates refer to age, sex, head motion, and years of education. D, Visualization of the GLM result by showing a scatter plot of E/I ratio residuals (after regressing out covariates) with respect to age (after regressing out covariates). Here, covariates refer to log‐transformed CSF Aβ42, sex, head motion, and years of education. Aβ, amyloid beta; ADNI, Alzheimer's Disease Neuroimaging Initiative; CN, cognitively normal; CSF, cerebrospinal fluid; E/I, excitation–inhibition; FD, framewise displacement; GLM, general linear model; MCI, mild cognitive impairment; SD, standard deviation; SE, standard error

To evaluate the robustness of these findings, we conducted three additional control analyses. First, we repeated the analysis using E/I ratio without ComBat harmonization (Table S5 in supporting information). Second, the GLM was refitted with an expanded set of covariates to account for common AD risk factors,^55^ including BMI, hypertension, diabetes, and smoking history, in addition to the original covariates (Table S6 in supporting information). Third, CSF Aβ42 was replaced with the CSF Aβ42/40 ratio (Table S7 in supporting information). All control analyses yielded similar results to the main analyses.

To characterize the model‐estimated components underlying the E/I ratio, we examined the temporally averaged excitatory activity *S_E_* and inhibitory activity *S_I_* separately (Tables S8 and S9 in supporting information). Lower CSF Aβ42 was associated with lower estimated *S_I_*, whereas its association with estimated *S_E_* was not statistically significant. Thus, within the pFIC model outputs, the higher E/I ratio associated with greater amyloid abnormality appeared more consistent with lower *S_I_* than with higher *S_E_*. However, because *S_E_* and *S_I_* are jointly estimated from the model, this pattern does not establish that reduced biological inhibition is the underlying mechanism (see section 4).

### 3.2 E/I ratio is more sensitive to CSF Aβ42 in sensory–motor cortex

In the previous section, we observed a negative association between E/I ratio and CSF Aβ42. To investigate whether this association varied across cortical regions, the same analysis was performed but for each cortical region independently. The spatial distribution of the regional regression coefficient (slope) is shown on the cortical surface in Figure 3A. All regions exhibited negative slopes that survived FDR correction (*q* < 0.05), indicating a global increase in E/I ratio with decreasing CSF Aβ42. Notably, the magnitude of this association varied systematically across the cortex and followed a clear network‐level structure. Specifically, lower order sensory–motor networks, including the somatomotor and visual networks, showed more pronounced negative slopes compared to higher order association networks (Figure 3B,C). This pattern followed the SA axis^53^ (Figure 3D,E).

**FIGURE 3:**
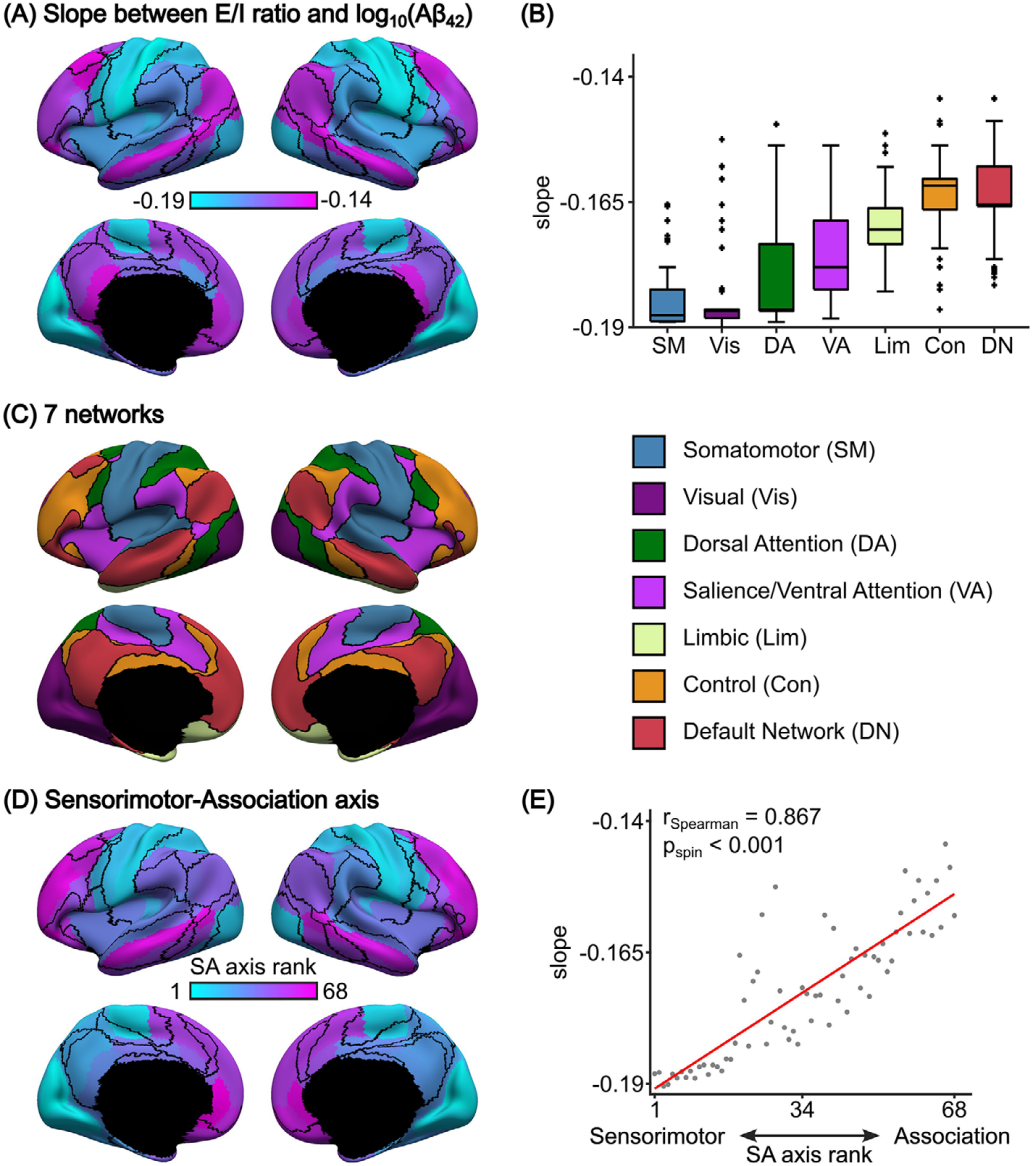
Spatial distribution of the associations between E/I ratio and CSF Aβ42. A, General linear model between regional cortical E/I ratio and log‐transformed CSF Aβ42, adjusting for age, sex, head motion (i.e., FD), and years of education. All regression coefficients (slopes) were negative and statistically significant (FDR *q* < 0.05). B, The slopes exhibited a spatial structure with lower‐order somatomotor and visual networks showing the largest (most negative) slopes. The boxes show the IQR and the median. Whiskers indicate 1.5 IQR. Black crosses represent outliers. C, Seven resting‐state networks^96^ are shown for reference. D, ROI rankings based on the sensorimotor–association axis (SA axis).^53^ E, Agreement between the E/I ratio–CSF Aβ42 slope (A) and SA axis rank. Spearman correlation *r* = 0.867, two‐tailed spin test *P* < 0.001. Aβ, amyloid beta; CSF, cerebrospinal fluid; E/I, excitation–inhibition; FD, framewise displacement; FDR, false discovery rate; IQR, interquartile range; ROI, region of interest

One possible explanation for the stronger association in sensory–motor cortex is the regional progression of tau pathology. Neurofibrillary tau pathology accumulates in association cortex from early Braak stages, whereas primary sensory and motor regions remain relatively spared until Braak V/VI.^56^, ^57^ Associated synaptic loss and neurodegeneration may suppress neuronal excitability,^58^, ^59^ attenuating the amyloid–E/I association in association cortex. To explore this explanation, we repeated the analyses in amyloid‐negative (*N* = 164) and amyloid‐positive (*N* = 122) participants, defined by global AV45 PET standardized uptake value ratio > 1.11.^60^, ^61^ None of the parcel‐specific associations was statistically significant, potentially due to the smaller sample sizes and restricted within‐group CSF Aβ42 range. Therefore, these results could not determine whether amyloid positivity amplified the SA contrast.

A complementary explanation is that Aβ preferentially affects parvalbumin (PVALB)‐expressing inhibitory interneurons^62^, ^63^ and PVALB expression is higher in sensory–motor cortex.^64^ We therefore tested whether regional slope magnitude correlated with cortical PVALB expression from the Allen Human Brain Atlas. The correlation was positive but not statistically significant (*r* = 0.359; *p* = 0.202; Figure S10 in supporting information), providing directional but not statistically reliable support for this explanation.

Similar results were obtained when using E/I ratio without ComBat harmonization (Figure S11 in supporting information) or with an expanded set of covariates including BMI, hypertension, diabetes, and smoking history (Figure S12 in supporting information). When replacing CSF Aβ42 with the CSF Aβ42/40 ratio, the regional slopes remained negative across all regions, with effect sizes comparable to those observed using CSF Aβ42 alone, and preserved the same network pattern. However, none of the regional associations survived FDR correction, likely due to reduced statistical power resulting from the smaller sample size with available CSF Aβ40 data (Figure S13 in supporting information).

### 3.3 E/I ratio partially mediates the association between CSF Aβ42 and cognition

Given that E/I ratio was associated with CSF Aβ42, we next asked whether E/I ratio was also linked to cognitive performance and whether it mediated the relationship between Aβ and cognition. To this end, we performed a mediation analysis^65^ with the ADSP PHC^54^ memory score as the dependent variable, log‐transformed CSF Aβ42 as the independent variable, and mean cortical E/I ratio as the mediator (Figure 4A). Age, sex, FD, and education were included as covariates in all pathways, and all continuous variables were *z* scored prior to analysis for interpretability.

**FIGURE 4:**
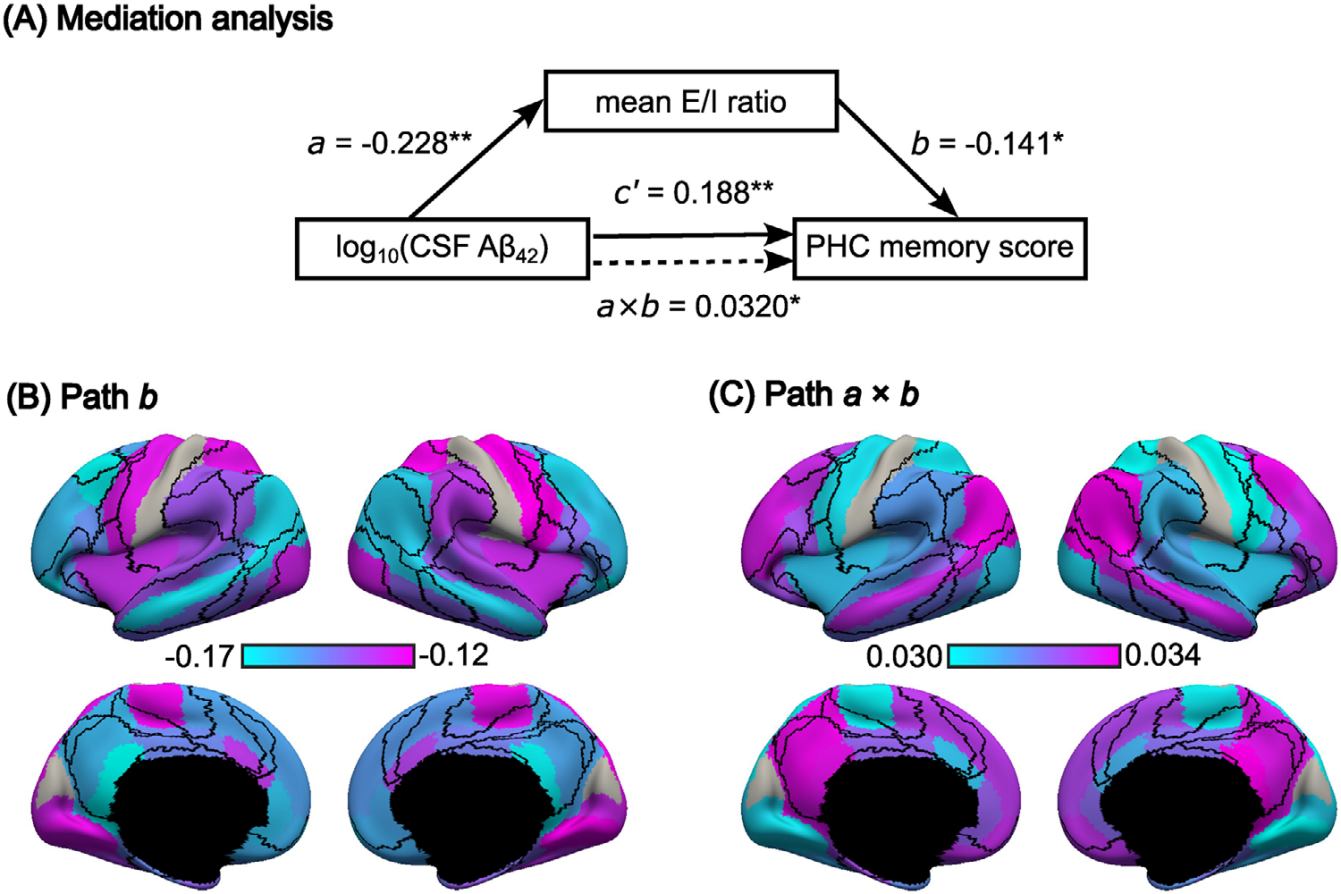
Mediation analyses linking CSF Aβ42, E/I ratio, and cognition. A, Schematic of the mediation analysis results, testing whether mean cortical E/I ratio mediates the association between log‐transformed CSF Aβ42 and cognitive performance (PHC memory score). Standardized regression coefficients are shown for path *a* (CSF Aβ42 → E/I ratio), path *b* (E/I ratio → cognition), and the direct effect *c′* (CSF Aβ42 → cognition, controlling for E/I ratio). All paths were adjusted for age, sex, head motion, and years of education. All continuous variables were *z* scored across participants. The *P* values were obtained from 10,000 bootstrap samples. * indicates *P* < 0.05. ** indicates *P* < 0.01. The proportion mediated (ratio of indirect to total effects) was 0.032/(0.032+0.188) = 14.5%. B, Regional standardized coefficients for path *b* (regional E/I ratio → cognition). C, Regional indirect effects (*a* *×* *b*). Regions that did not survive false discovery rate correction across 68 cortical regions (*q* < 0.05) are shown in gray. Aβ, amyloid beta; CSF, cerebrospinal fluid; E/I, excitation–inhibition; PHC, Phenotype Harmonization Consortium

Consistent with the GLM results, CSF Aβ42 was negatively associated with mean E/I ratio (path *a*), indicating higher E/I ratio with greater amyloid burden (i.e., lower CSF Aβ42). Mean E/I ratio was, in turn, negatively associated with PHC memory performance when controlling for CSF Aβ42 (path *b*), such that higher E/I ratio was related to poorer cognition, in line with prior work linking E/I imbalance to cognitive dysfunction.^66^, ^67^ CSF Aβ42 remained positively associated with cognition after accounting for E/I ratio (path *c′*). The indirect effect of CSF Aβ42 on cognition mediated through E/I ratio (*a × b*) was statistically significant, and accounted for ≈ 14.5% of the total effect. This suggests that alterations in E/I ratio partially explain the association between amyloid pathology and memory performance.

At the regional level, the association between E/I ratio and memory performance (path *b*) was the strongest in association cortex (Figure 4B). A similar spatial pattern was observed for the regional indirect effects (*a × b*), which were also strongest in association regions (Figure 4C). Together, these findings suggest that while sensory–motor regions exhibit heightened sensitivity of E/I ratio to Aβ (Figure 3A), the downstream coupling between E/I ratio and cognitive performance is stronger in association cortices, especially in the default network, which is known to support memory function.^68^

To evaluate the robustness of the mediation findings, we repeated mediation analyses at both the mean E/I and regional level. First, we repeated using E/I ratio without ComBat harmonization (Figure S14 in supporting information). Second, we included an expanded set of covariates additionally adjusting for BMI, hypertension, diabetes, and smoking history (Figure S15 in supporting information). Third, we replaced CSF Aβ42 with CSF Aβ42/40 ratio (Figure S16 in supporting information). Across all control analyses, the indirect effect through the mean E/I ratio was significant. The regional estimates of path *b* also remained significant and exhibited spatial patterns consistent with the main results. The regional indirect effects (*a × b*) were also significant and spatially consistent for both the non‐harmonized E/I ratio and the expanded covariate models. However, when using the CSF Aβ42/40 ratio, none of the regional indirect effects survived FDR correction, despite effect sizes comparable to those observed with CSF Aβ42 alone.

### 3.4 Plasma p‐tau217 is positively associated with mean E/I ratio

Having established that the E/I ratio is associated with CSF Aβ42 and cognition in the ADNI dataset, we next turned to plasma‐based biomarkers. Among these, plasma p‐tau217 has been shown to outperform other plasma AD biomarkers in detecting Aβ pathology, including p‐tau181 and p‐tau231.^31^, ^69^ Here, we used resting‐state fMRI data and plasma p‐tau217 measurements from the MACC Harmonization dataset^41^ to examine the relationship between E/I ratio and blood‐based biomarker of AD pathophysiology (*N* = 240). Participant demographics are summarized in Figure 5A.

**FIGURE 5:**
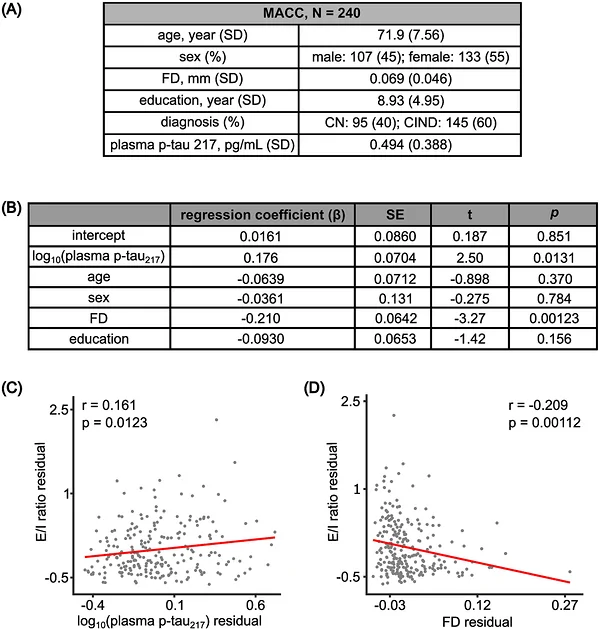
Association between mean cortical E/I ratio and plasma p‐tau217. A, Participant characteristics in the MACC dataset. B, GLM between mean cortical E/I ratio and log‐transformed plasma p‐tau217, adjusting for age, sex (female = 0, male = 1), head motion (i.e., FD), and years of education. An intercept term is included. All continuous variables were *z* scored prior to model fitting. C, Visualization of the GLM result by showing a scatter plot of E/I ratio residuals (after regressing out covariates) with respect to log‐transformed plasma p‐tau217 residuals (after regressing out covariates). Here, covariates refer to age, sex, head motion, and years of education. D, Visualization of the GLM result by showing a scatter plot of E/I ratio residuals (after regressing out covariates) with respect to FD (after regressing out covariates). Here, covariates refer to log‐transformed plasma p‐tau217, age, sex, and years of education. Aβ, amyloid beta; CN, cognitively normal; CSF, cerebrospinal fluid; E/I, excitation–inhibition; FD, framewise displacement; GLM, general linear model; MACC, Memory, Ageing and Cognition Centre; MCI, mild cognitive impairment; p‐tau, phosphorylated tau; SD, standard deviation; SE, standard error

In contrast to CSF Aβ42, plasma p‐tau217 levels increase with AD progression.^70^, ^71^ Importantly, a growing body of evidence suggests that elevated plasma p‐tau217 reflects Aβ pathophysiology in the early disease stages, preceding tau aggregation.^72^, ^73^ Thus, plasma p‐tau217 provides a complementary biomarker of amyloid pathophysiology to CSF Aβ42 and may share similar neurobiological mechanisms involving altered E/I ratio. To test this hypothesis, we fitted a GLM between mean E/I ratio and plasma p‐tau217, adjusting for the same covariates as the corresponding ADNI analysis.

Regression results are reported in Figure 5B. Similar to CSF Aβ42, greater amyloid pathophysiology (i.e., higher p‐tau217) was associated with higher E/I ratio (Figure 5B,C). However, unlike in ADNI, age was not a significant predictor of E/I ratio in the MACC cohort, whereas lower head motion was associated with higher E/I ratio (Figure 5B,D). Therefore, the association between p‐tau217 and E/I ratio cannot be explained by individuals with higher disease burden moving more in the MRI scanner.

To evaluate robustness, we conducted two additional control analyses. First, we refitted the GLM using an expanded covariate set that additionally included BMI, hypertension, diabetes, and smoking history (Table S17 in supporting information). Second, plasma p‐tau217 was replaced with plasma p‐tau181 (Table S18 in supporting information). The control analyses yielded similar results to the main analysis.

We also examined excitatory activity *S_E_* and inhibitory activity *S_I_* separately (Tables S19 and S20 in supporting information). Plasma p‐tau217 was significantly associated with *S_I_* but not with *S_E_*. As in ADNI, the higher E/I ratio associated with greater biomarker abnormality therefore appeared more consistent with lower *S_I_* than with higher *S_E_* within the pFIC model outputs. However, *S_E_* and *S_I_* are jointly estimated simulated quantities and should not be interpreted as independent biological measurements (see section 4).

### 3.5 E/I ratio is more sensitive to plasma p‐tau217 in sensory–motor cortex

Similar to the regional analysis between E/I ratio and CSF Aβ42 (Figure 3), we next examined regional relationships between E/I ratio and plasma p‐tau217. For each cortical region, we fitted a GLM with regional E/I ratio as the dependent variable and log‐transformed plasma p‐tau217 as the independent variable, adjusting for the same covariates as the corresponding ADNI analysis. The spatial distribution of the resulting regional regression coefficients is shown on the cortical surface in Figure 6A.

**FIGURE 6:**
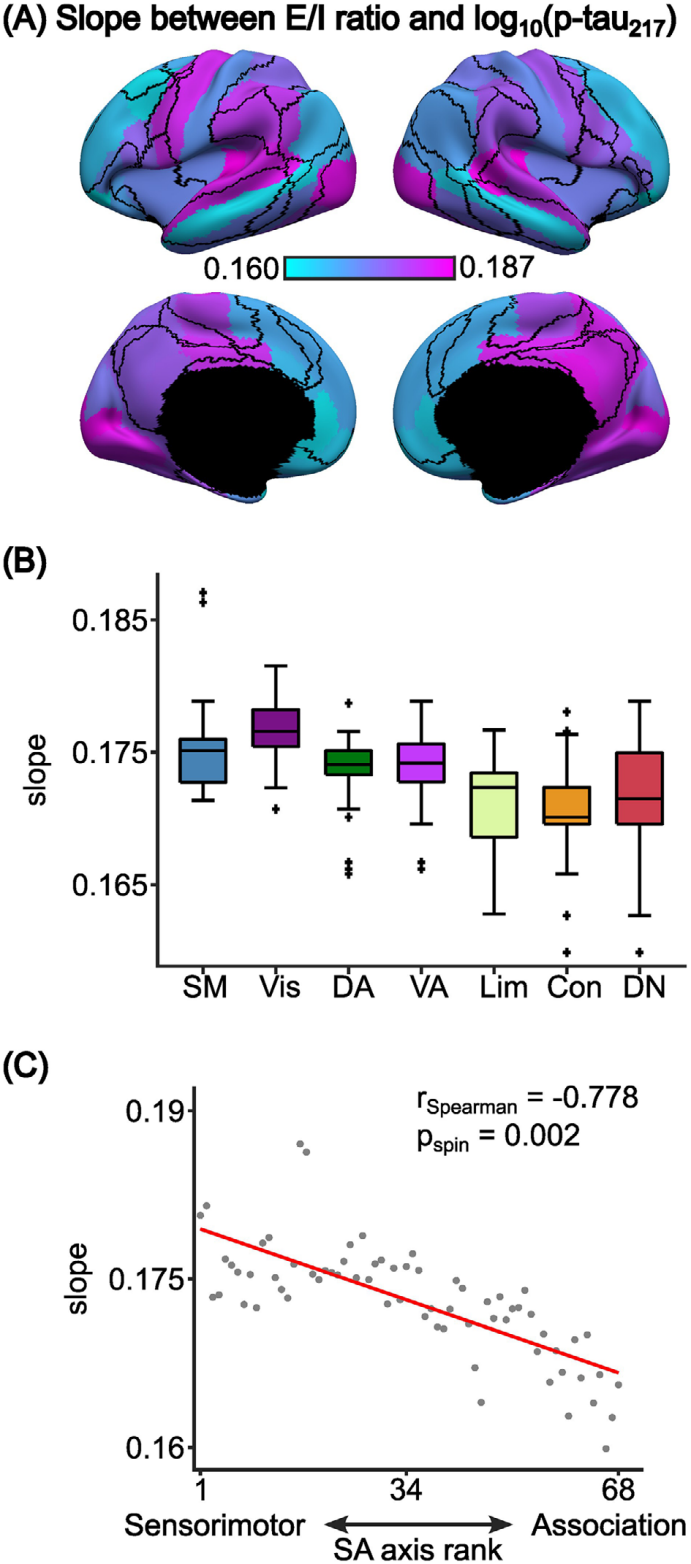
Spatial distribution of the associations between E/I ratio and plasma p‐tau217. A, General linear model between regional cortical E/I ratio and log‐transformed plasma p‐tau217, adjusting for age, sex, head motion (i.e., FD), and years of education. All regression coefficients (slopes) were positive and statistically significant (FDR *q* < 0.05). B, The slopes exhibited a spatial structure with lower order somatomotor and visual networks showing the largest (most positive) slopes. The boxes show the IQR and the median. Whiskers indicate 1.5 IQR. Black crosses represent outliers. C, Agreement between the E/I ratio–plasma ptau217 slope (A) and SA axis rank. Spearman correlation *r* = −0.778, two‐tailed spin test *p* = 0.002. E/I, excitation–inhibition; FD, framewise displacement; FDR, false discovery rate; IQR, interquartile range; p‐tau, phosphorylated tau; ROI, region of interest; SA, sensorimotor–association

Across the cortex, all regional slopes were positive, indicating that higher plasma p‐tau217 levels were associated with higher E/I ratios. This directionality contrasts with the negative slopes observed for CSF Aβ42 in ADNI and is consistent with the neurophysiology of these biomarkers along the AD progression. Similar to CSF Aβ42, the strongest associations were again observed in lower order sensory–motor regions, with weaker effects in higher order association cortex (Figure 6B). This spatial pattern closely followed the SA axis^53^ (Figure 6C), again suggesting heightened sensitivity of the sensory–motor cortex to global amyloid pathophysiology.

Given the similar regional patterns in ADNI and MACC, we explored whether the same staging‐ and PVALB‐related explanations were compatible with the MACC findings. We repeated the regional analyses in amyloid‐negative (*N* = 195) and amyloid‐positive (*N* = 45) participants, using a p‐tau217 cutoff of 0.471.^31^ None of the parcel‐specific associations was statistically significant, potentially due to the smaller sample sizes and restricted within‐group p‐tau217 range. We also tested whether regional slope magnitude correlated with cortical PVALB expression. The correlation was positive but non‐significant (*r* = 0.403; two‐sided spin‐test *p* = 0.116; Figure S21 in supporting information), providing directional but not statistically reliable support for this explanation.

Consistent results were obtained when refitting the regional models with an expanded covariate set, additionally including BMI, hypertension, diabetes, and smoking history (Figure S22 in supporting information). However, replacing plasma p‐tau217 with plasma p‐tau181 yielded no regions with significant associations after FDR correction although the regional slopes remained positive across all regions (Figure S23 in supporting information).

### 3.6 E/I ratio partially mediates the association between plasma p‐tau217 and cognition

Finally, we examined whether the E/I ratio mediates the association between plasma p‐tau217 and cognitive performance. To derive a similar metric of cognitive ability used in the ADNI mediation analysis, cognitive ability in the MACC dataset was derived using a memory score aimed to capture analogous memory‐related domains. This memory score was constructed from selected MMSE subscores and verbal memory measures (see Table S4 for the included measures and their factor loadings).

Mediation analysis was performed with plasma p‐tau217 as the independent variable, mean E/I ratio as the mediator, and the memory score as the dependent variable, while adjusting for the same covariates as the corresponding ADNI analysis (Figure 7A). Plasma p‐tau217 showed a significant positive association with mean E/I ratio (*a* = 0.176), while higher E/I ratio was significantly associated with worse memory performance (*b* = −0.120). Plasma p‐tau217 also exhibited a significant direct association with memory after accounting for E/I ratio (*c′* = −0.391). The indirect effect of plasma p‐tau217 on cognition through E/I ratio was significant (*a × b* = −0.0211) and explained 5.1% of the total effect, indicating a partial mediation.

**FIGURE 7:**
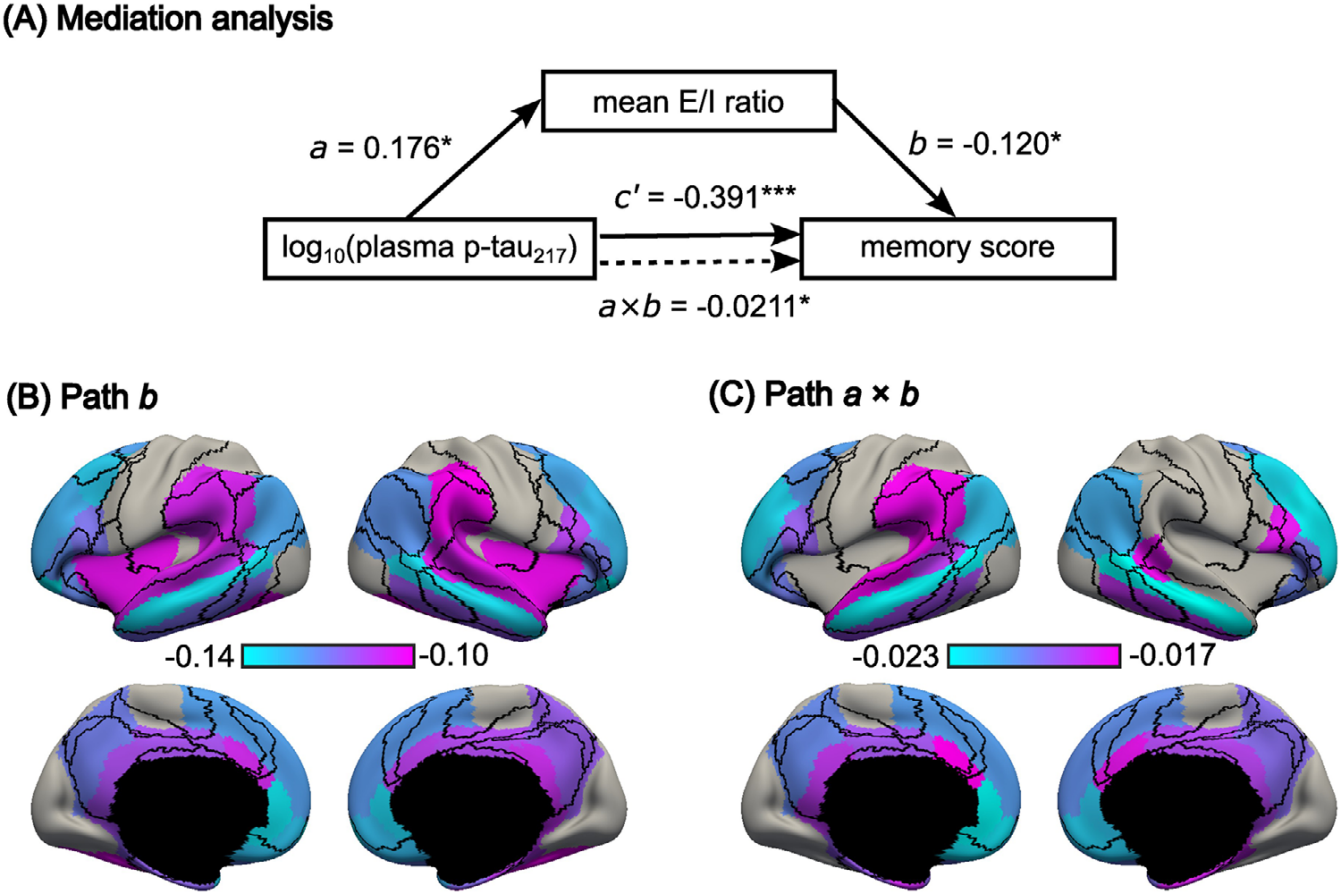
Mediation analyses linking plasma p‐tau217, E/I ratio, and cognition. A, Schematic of the mediation analysis results, testing whether mean cortical E/I ratio mediates the association between log‐transformed p‐tau217 and cognitive performance (memory score). Standardized regression coefficients are shown for path *a* (plasma p‐tau217 → E/I ratio), path *b* (E/I ratio → cognition), and the direct effect *c′* (plasma p‐tau217→ cognition, controlling for E/I ratio). All paths were adjusted for age, sex, head motion, and years of education. All continuous variables were *z* scored across participants. *P* values were obtained from 10,000 bootstrap samples, * indicates *P* < 0.05; *** indicates *P* < 0.001. The proportion mediated (ratio of indirect to total effects) was 0.0211/(0.0211+0.391) = 5.1%. B, Regional standardized coefficients for path *b* (regional E/I ratio → cognition). C, Regional indirect effects (*a × b*). Regions that did not survive false discovery rate correction across 68 cortical regions (*q* < 0.05) are shown in gray. E/I, excitation–inhibition; p‐tau, phosphorylated tau

At the regional level, the association between E/I ratio and memory performance (path *b*) was the strongest in association cortex (Figure 7B), paralleling results in the ADNI cohort (Figure 4B). Consequently, regional indirect effects (*a × b*) were also the strongest in association regions (Figure 7C). Together, these findings suggest that the mediating role of E/I ratio is spatially structured, with higher order association cortices contributing more prominently to the indirect effect of plasma p‐tau217 on cognition, mirroring the regional mediation effect observed for CSF Aβ42 in ADNI.

As control analyses, the mediation analysis was first repeated with an expanded covariate set that additionally included BMI, hypertension, diabetes, and smoking history. While global indirect effects (*a × b*) and regional path *b* coefficients remained significant within the association cortices, none of the regional indirect effects (*a × b*) reached statistical significance (Figure S24 in supporting information). Second, plasma p‐tau217 was replaced with plasma p‐tau181 as the independent variable. Both the global and regional mediation results remained consistent, with significant indirect effects through mean E/I ratio and regional path *b* and (*a × b*) patterns (Figure S25 in supporting information).

Finally, we assessed the sensitivity of the mediation findings to how memory was operationalized. We first repeated the analyses using each constituent measure of the MACC memory composite and the closest corresponding ADNI measure (Table S26 in supporting information). Across all measures in both cohorts, paths *a*, *b*, and *c′* and the indirect effect (*a × b*) retained the same signs as in the primary composite‐score analyses, although their magnitudes and statistical support varied. The global indirect effect remained significant only for Story Recall in MACC, and for none of the corresponding ADNI measures after FDR correction.

We next constructed an ADNI composite more closely aligned with the MACC composite in content and weighting by applying the MACC factor loadings to the corresponding ADNI measures (Table S27 in supporting information). With this matched composite, paths *a*, *b*, and *c′* and the indirect effect retained their original signs, but path *b* and the indirect effect were attenuated, and the indirect effect was no longer statistically significant (Figure S28 in supporting information). At the regional level, path *b* and the indirect effects remained strongest in higher order association cortices, but no regional effects survived FDR correction. The estimated proportion mediated decreased from 14.5% to 12.4% but remained larger than the 5.1% estimated in MACC. Overall, the directions of the memory‐dependent paths were consistent across alternative operationalizations of memory, whereas their magnitudes and statistical support depended on measurement choice.

## 4 DISCUSSION

In this study, we investigated how E/I ratio relates to AD biomarkers and memory performance across non‐demented participants from two independent cohorts. Higher cortical E/I ratio was associated with worse memory performance and with molecular markers of AD pathophysiology, indexed by lower CSF Aβ42 in ADNI and higher plasma p‐tau217 in MACC. Mediation analyses further demonstrated that E/I ratio partially mediated the relationship between both biomarkers and cognition, suggesting that E/I imbalance represents a potential mechanistic link between molecular pathology and cognition. Importantly, these findings were consistent across distinct biomarker modalities and cohorts, supporting the robustness and generalizability of the observed associations.

One key finding of this study is that elevated E/I ratio was associated with poorer memory performance. The effect was especially pronounced in the default network, which is known to support memory function^68^ and strongly implicated in AD.^74^ From a circuit perspective, memory, particularly episodic memory, relies on precise temporal coordination of neural firing within hippocampal‐cortical networks.^75^ Excessive excitation or insufficient inhibition can degrade this coordination, leading to increased neural noise, reduced signal‐to‐noise ratio, and impaired synaptic plasticity.^66^, ^76^ Hyperexcitability may disrupt memory formation by interfering with long‐term potentiation and promoting aberrant network synchronization.^77^, ^78^ Moreover, sustained elevations in excitation can induce homeostatic stress on neural circuits, rendering them less flexible and less capable of supporting adaptive cognitive processes.^79^ Taken together, these mechanisms provide a plausible explanation for why higher E/I ratio, reflecting a shift toward excitation‐dominant dynamics, is associated with worse memory performance in non‐demented individuals.

The observed association between CSF Aβ42 and cortical E/I ratio provides insight into the circuit‐level consequences of early amyloid pathophysiology. Reduced CSF Aβ42 is widely interpreted as a marker of increased global amyloid deposition in the brain.^80^, ^81^, ^82^, ^83^ Consistent with prior work demonstrating that Aβ enhances excitatory transmission while disrupting inhibitory synaptic efficacy,^84^ we observed a negative association between CSF Aβ42 and mean cortical E/I ratio, indicating that increasing amyloid burden is associated with a shift toward excessive neuronal excitation relative to inhibition.

The biomarker–E/I association was stronger in sensory–motor than association cortex, despite amyloid accumulation in early AD typically being greatest in association cortex.^85^ Two non‐mutually exclusive mechanisms could contribute to this pattern. First, tau pathology reaches association cortex earlier than primary sensory–motor cortex^56^, ^57^ and the resulting synaptic loss and neurodegeneration may attenuate amyloid‐related increases in E/I ratio. Second, Aβ may preferentially impair PVALB‐positive inhibitory interneurons^62^, ^63^ and higher PVALB expression in sensory–motor cortex^64^ may increase its vulnerability to disruption of inhibitory control. However, the amyloid‐stratified analyses were too imprecise to evaluate the first explanation, and the spatial correlations with PVALB expression were positive but non‐significant. These mechanisms therefore remain plausible but unconfirmed.

In the independent Singapore‐based Asian MACC cohort, higher plasma p‐tau217 was likewise associated with higher estimated E/I ratio. Plasma p‐tau217 reflects amyloid‐responsive soluble tau pathophysiology and, particularly when AD‐type tau pathology is present, may also capture tau burden.^72^, ^86^ Nevertheless, the direction of the association was consistent with the ADNI finding that greater biomarker abnormality was associated with higher estimated E/I ratio.

The principal associations therefore showed a consistent directional pattern across the North American ADNI cohort and the Singapore‐based Asian MACC cohort, despite their different geographical, ethnic, and sociocultural contexts and the use of different biomarker modalities. This cross‐cohort consistency supports the robustness of the broad pattern beyond a single predominantly North American setting and broadens its potential generalizability among non‐demented older adults. However, the strengths of the component associations and the estimated proportions mediated differed between cohorts. The convergence should therefore be interpreted as consistency in the overall pattern rather than equivalence of effect sizes.

The mediation analyses were consistent with E/I ratio representing one circuit‐level component of the association between AD biomarkers and memory performance. However, the estimated indirect effect accounted for only a minority of the total effect—14.5% in ADNI and 5.1% in MACC. Our framework should therefore be understood as a partial, circuit‐level account of the biomarker–memory association rather than a comprehensive or causal explanation. Other processes, including tau propagation,^60^ synaptic loss,^84^ and cerebrovascular disease,^41^ may contribute independently or interact with E/I‐related circuit alterations.

Furthermore, the proportion of the biomarker–memory association mediated by E/I ratio was smaller in MACC than in ADNI (5.1% vs. 14.5%). As shown in Figures 4 and 7, this difference reflected both modestly weaker biomarker–E/I (path *a*) and E/I–memory associations (path *b*), as well as a substantially stronger biomarker–memory association outside the modeled E/I pathway (path *c’*) in MACC. Several cohort differences may contribute. First, CSF Aβ42 is a relatively proximal marker of cerebral amyloid dysmetabolism, whereas plasma p‐tau217 reflects amyloid‐responsive soluble tau pathophysiology and, when AD‐type tau pathology is present, may also capture tau burden.^72^, ^86^ This is relevant even before dementia because tau pathology can occur in amyloid‐positive cognitively unimpaired and MCI individuals.^87^ Tau‐related effects on cognition outside the modeled E/I pathway could strengthen the remaining biomarker–memory association, while potentially opposing amyloid‐ and tau‐related associations with E/I ratio could weaken the indirect pathway.

The MACC cohort also had a higher cerebrovascular burden than ADNI: mean log[WMH/ICV]—logarithm of the ratio of white matter hyperintensity volume and intracranial volume—was −5.75 versus −6.81 (*P* < 0.001). Cerebrovascular disease is an established contributor to cognitive impairment.^44^, ^88^, ^89^ However, additional adjustment for WMH/ICV left the MACC mediation estimates virtually unchanged, including the proportion mediated (5.0% after adjustment versus 5.1% in the primary analysis). Measured WMH burden therefore did not explain the smaller proportion mediated in MACC, although contributions from other cerebrovascular abnormalities cannot be excluded.

Cognitive measurement also affected the mediation estimates. Across the individual constituent measures, the paths retained the same directions as in the primary analyses, but the indirect effect was significant only for Story Recall in MACC. Although we did not directly compare reliability, composites may provide more stable estimates by reducing measure‐specific error, potentially explaining the weaker statistical evidence for individual measures. With an ADNI composite more closely matched to MACC, the indirect effect was attenuated and no longer significant, while the proportion mediated decreased from 14.5% to 12.4% but remained larger than the 5.1% observed in MACC. Composite construction may therefore affect the estimates but did not fully explain the between‐cohort difference. Nevertheless, differences in how these tests function across educational, linguistic, cultural, and other sociodemographic contexts may also have contributed to the differing mediation estimates between cohorts.

Taken together, biomarker biology, cerebrovascular burden, cognitive measurement, and other cohort characteristics may all contribute to the differing mediation estimates. Because these factors are partially confounded with cohort membership, their individual contributions cannot be determined from the present analyses. The cross‐cohort findings therefore provide stronger evidence for consistency in the direction of the associations than for equivalence in the strength of the indirect pathway.

Several limitations warrant consideration. First, our study did not include patients with clinically diagnosed AD dementia. Later stage AD involves complex and interacting processes, including widespread tau pathology, synaptic loss, neuronal death, and network disintegration, making it challenging to formulate clear hypotheses about how E/I ratio evolves over the full disease course.^16^ By focusing on non‐demented populations, we aimed to identify early circuit‐level changes that may precede wide‐spread neurodegenerative alterations. Additionally, the available sample size for some biomarkers was limited, which may reduce statistical power and affect the detection of regional associations. Second, the estimated E/I ratio is an aggregate model‐derived measure. Although the separate analyses showed associations with estimated inhibitory but not excitatory activities in both cohorts, the model may not uniquely distinguish their respective contributions to the E/I ratio. The apparent predominance of inhibition should consequently be interpreted as a feature of the model estimates rather than evidence that reduced biological inhibition is the primary mechanism or support for a particular therapeutic strategy. In addition, the E/I ratio is calculated as a temporal average over the entire resting‐state fMRI session, which improves stability but does not capture moment‐to‐moment fluctuations. Third, the biophysical modeling does not explicitly incorporate non‐neuronal cell types, such as astrocytes and microglia, which are increasingly recognized as active contributors to synaptic regulation and AD pathology.^90^, ^91^ Glial cells play critical roles in Aβ clearance, synaptic modulation, and neuroinflammation, all of which may influence E/I ratio.^92^

Future work should extend the current framework to individuals with more advanced disease stages. Longitudinal studies combining fMRI‐based E/I estimation with CSF and plasma biomarker measurements will be particularly valuable for characterizing how circuit dynamics evolve over time and for testing whether E/I imbalance predicts subsequent cognitive decline. Complementary measurements sensitive to glutamatergic and GABAergic neurochemistry, such as magnetic resonance spectroscopy, may help distinguish excitatory from inhibitory contributions, while time‐resolved modeling approaches could characterize their temporal dynamics. Incorporating glial mechanisms and multimodal imaging data may further enhance the biological plausibility of the systems‐level pathways linking molecular pathology to cognition.

In summary, our study provides convergent evidence that altered E/I ratio represents a partial circuit‐level mechanism linking AD biomarkers and cognitive performance in non‐demented populations. By relating accessible CSF and plasma markers to in vivo estimates of neuronal dynamics, our findings offer new insights into how amyloid‐related disruptions of the E/I ratio may contribute to cognitive impairment. These early disruptions of neuronal dynamics may represent a potentially modifiable therapeutic target for mitigating the downstream effects of AD pathology on cognitive function.

## CONFLICT OF INTEREST STATEMENT

The authors declare no conflicts of interest. Author disclosures are available in the Supporting Information.

## CONSENT STATEMENT

The ADNI study was approved by the institutional review boards (IRBs) of all participating institutions with informed written consent from all participants at each site. Approval for the MACC Harmonization study was obtained from the Singapore National Healthcare Group Domain‐Specific Review Board and written informed consent was obtained for all participants prior to study commencement.

## Supporting information




**Supporting Information**: alz71891‐sup‐0001‐SuppMat.docx

**Supporting Information**: alz71891‐sup‐0002‐ICMJE.pdf
