# Supplementary material for: Excitation–inhibition imbalance links amyloid pathophysiology to cognition in non‐demented individuals
Source: Alzheimers Dement. 2026 Sep 30;22(10):e71891. doi: 10.1002/alz.71891 (PMC13624711; doi:10.1002/alz.71891)
Supplement: Supplementary file 2 [file ALZ-22-e71891-s002.docx]

SUPPLEMENTARY INFORMATION

**Supplementary Methods**

## **S1. Functional connectivity dynamics (FCD) computation**

Functional connectivity dynamics (FCD) matrices were computed as follows. We defined a window with a length of 60s as recommended by previous studies (Leonardi & Van De Ville, 2015; Liégeois et al., 2017). The window was moved from the first frame to the k-th frame of fMRI time series, resulting in k sliding windows in total, where k = T - ⌊T/60⌋ + 1 and ⌊x⌋ represents the floor function. FC was computed within each of k sliding windows. Each sliding window FC matrix was then vectorized by only considering the upper triangular entries. The vectorized FC matrices were correlated with each other generating a k × k FCD matrix.

## **S2. Parametric feedback inhibition control (pFIC) model**

The details of the model can be found in our previous study (Zhang et al., 2024). Here, we provide some intuition for the pFIC model. We start with the formulation of the original feedback inhibition control (FIC) model (Deco et al., 2014). Neuronal activities of the $j$-th cortical region follow the nonlinear differential equations shown below

$$I_{j}^{(E)}=W_{E}I_{0}+w_{EE}J_{NMDA}S_{j}^{(E)}+GJ_{NMDA}\sum_{k} C_{jk}S_{k}^{(E)}-w_{IE}S_{j}^{(I)} (1)$$

$$I_{j}^{(I)}=W_{I}I_{0}+w_{EI}J_{NMDA}S_{j}^{(E)}-w_{II}S_{j}^{(I)} (2)$$

$$r_{j}^{(E)}=\phi\left( I_{j}^{\left( E \right)} \right)=\frac{a_{E}I_{j}^{(E)}-b_{E}}{1-exp(-d_{E}\left( a_{E}I_{j}^{\left( E \right)}-b_{E} \right))} (3)$$

$$r_{j}^{(I)}=\phi\left( I_{j}^{\left( I \right)} \right)=\frac{a_{I}I_{j}^{(I)}-b_{I}}{1-exp(-d_{I}\left( a_{I}I_{j}^{\left( I \right)}-b_{I} \right))} (4)$$

$$\frac{dS_{j}^{(E)}}{dt}=-\frac{S_{j}^{\left( E \right)}}{\tau_{E}}+\left( 1-S_{j}^{\left( E \right)} \right)\gamma r_{j}^{\left( E \right)}+\sigma\nu_{j}(t) (5)$$

$$\frac{dS_{j}^{(I)}}{dt}=-\frac{S_{j}^{\left( I \right)}}{\tau_{I}}+r_{j}^{\left( I \right)}+\sigma\nu_{j}(t) (6)$$

where $S$, $r$, and $I$ represent synaptic gating variables, firing rate, and synaptic currents respectively. The superscripts $E$ and $I$ denote the excitatory and inhibitory neuronal populations respectively.

The input current $I_{j}^{(E)}$ of the excitatory population of the $j$-th cortical ROI is the sum of four inputs (Equation 1). The first input is the external input current $W_{E}I_{0}$, which might include subcortical delays. The second input is the intra-regional excitatory-to-excitatory current governed by the excitatory-to-excitatory recurrent connection strength $w_{EE}$ scaled by the synaptic coupling constant $J_{NMDA}$. The third input is the inter-regional input, which is controlled by the SC matrix ($C_{jk}$ is the connectivity between regions $j$ and $k$) and scaled by the global constant $G$. The fourth input is the intra-regional negative feedback from the inhibitory population governed by the inhibitory-to-excitatory connection strength $w_{IE}$.

The input current $I_{j}^{(I)}$ of the inhibitory population of the $j$-th cortical ROI is the sum of three inputs (Equation 2). The first input is the external input current $W_{I}I_{0}$. The second input is the intra-regional positive feedback from the excitatory population governed by the excitatory-to-inhibitory connection strength $w_{EI}$ scaled by the synaptic coupling constant $J_{NMDA}$. The third input is the intra-regional inhibitory-to-inhibitory current governed by the inhibitory-to-inhibitory recurrent connection strength $w_{II}$.

The excitatory input current $I_{j}^{(E)}$ and inhibitory input current $I_{j}^{(I)}$ are transformed into firing rates via the input-output functions specified in Equations 3 and 4. Following previous studies (Deco et al., 2014), the parameters of the input-output function were set to be $a_{E}=310$n/C, $a_{I}=615$n/C, $b_{E}=125$Hz, $b_{I}=177$Hz, $d_{E}=0.16$s and $d_{I}=0.087$s. Finally, the rate of change of the synaptic gating variables $S_{j}^{(E)}$ and $S_{j}^{(I)}$ are computed via equations 5 and 6. Following previous studies, the kinetic parameters for synaptic activities $\tau_{E}$, $\tau_{I}$and $\gamma$ were set to 100ms, 10ms and 0.641 respectively. $\nu_{j}\left( t \right)$ corresponds to uncorrelated standard Gaussian noise with the noise amplitude being controlled by $\sigma$.

Following the original study (Deco et al., 2014), $w_{II}$, $W_{E}$, $W_{I}$, $I_{0}$ and $J_{NMDA}$ were set to 1, 1, 0.7 0.382nA and 0.15nA respectively in the current study. The inhibitory-to-excitatory connection strength $w_{IE}$was computed analytically to ensure that the excitatory firing rate is maintained to be around 3Hz (Demirtaş et al., 2019). We note that this analytical computation assumes a noiseless system, so in practice, we imposed a constraint that the firing rate is between 2.7Hz to 3.3Hz. During the estimation of the pFIC model (next section), parameters were rejected if firing rates fall outside this range.

The excitatory-to-excitatory recurrent connection strength $w_{EE}$, excitatory-to-inhibitory connection strength $w_{EI}$, noise amplitude $\sigma$ and global SC scaling constant $G$ are unknown parameters, which will be estimated by fitting to empirical fMRI data (next section). To reduce the computational complexity, we reduced the number of “free” parameters by parameterizing the local synaptic parameters with a linear combination of the first principal FC gradient and T1w/T2w ratio map (Kong et al., 2021; Zhang et al., 2024). Here, we considered the same approach for the FIC model by parameterizing $w_{EE}$, $w_{EI}$, and $\sigma$ as a linear combination of T1w/T2w ratio map and first principal FC gradient.

$$w_{EE,j}=a+b\times{T1w/T2w}_{j}+c\times{FC gradient}_{j} (7)$$

$$w_{EI,j}=d+e\times{T1w/T2w}_{j}+f\times{FC gradient}_{j} (8)$$

$$\sigma_{j}=g+h\times{T1w/T2w}_{j}+i\times{FC gradient}_{j} (9)$$

where *j* denotes the *j*-th ROI. By adopting this parameterization approach, the number of “free” numbers was reduced to 3 × 3 + 1 = 10 parameters ($a, b, c, d, e,f,g, h,i, G$). We refer to this model as the pFIC model.

Given a fixed set of model parameters, Equations 1 to 6 can be used to simulate the time courses of excitatory and inhibitory synaptic gating variables ($S_{j}^{(E)}$ and $S_{j}^{(I)}$) of each ROI. The regional E/I ratio was defined as the ratio between the temporal average of $S_{j}^{(E)}$ and $S_{j}^{(I)}$ (i.e., $\left\langle S_{j}^{(E)} \right\rangle/\left\langle S_{j}^{(I)} \right\rangle$). The mean cortical E/I ratio was the average of regional E/I ratios across all cortical ROIs. The simulated excitatory synaptic gating variables ($S_{j}^{(E)}$) were also fed to the Balloon-Windkessel hemodynamic model to simulate fMRI BOLD signals (Stephan et al., 2007; Deco et al., 2014; Heinzle et al., 2016). The simulated fMRI BOLD signals were then used to generate simulated FC and FCD.

## **S3. Individual-level pFIC optimization and E/I ratio estimation**

The 10 unknown parameters of the pFIC model were optimized by maximizing fit to empirical static FC and FCD (Zhang et al., 2024). For the optimization algorithm, we used the covariance matrix adaptation evolution strategy (CMA-ES) (Hansen, 2006; Kong et al., 2021). The CMA-ES algorithm was iterated 100 times, yielding a total of 100 candidate parameter sets. The parameter set that achieved the lowest overall cost was selected as the optimal parameter set. During optimization, the temporal resolution of fMRI simulation was set as 6ms to speed up the computation. After selecting the optimal parameter set, to ensure a more accurate simulation, the temporal resolution was set as 0.5ms for E/I ratio estimation. Specifically, we ran the simulation with the optimal parameter set 1000 times with different noise instantiations. This resulted in 1000 sets of $\left\langle S^{(E)} \right\rangle$ and $\left\langle S^{(I)} \right\rangle$ , and the individual-level E/I ratio estimate was computed as the average across 1000 ratios of $\left\langle S^{(E)} \right\rangle$ and $\left\langle S^{(I)} \right\rangle$. As the fMRI data were collected from multiple scanning sites in the ADNI dataset, the estimated E/I ratios were harmonized for each ROI independently using ComBat across participants (Fortin et al., 2017). No additional site harmonization was required for the MACC dataset as the data were collected with a single scanner. Fitting the pFIC model to a single participant takes approximately 5 hours on one NVIDIA RTX 3090 GPU. Random seeds controlling the CMA-ES optimization are specified in the configuration files, enabling the stochastic components to be reproduced.

**Reference**

Anderson, K. M., Collins, M. A., Chin, R., Ge, T., Rosenberg, M. D., & Holmes, A. J. (2020). Transcriptional and imaging-genetic association of cortical interneurons, brain function, and schizophrenia risk. *Nature Communications*, *11*(1), 2889. https://doi.org/10.1038/s41467-020-16710-x

Deco, G., Ponce-Alvarez, A., Hagmann, P., Romani, G. L., Mantini, D., & Corbetta, M. (2014). How Local Excitation-Inhibition Ratio Impacts the Whole Brain Dynamics. *Journal of Neuroscience*, *34*(23), 7886–7898. https://doi.org/10.1523/JNEUROSCI.5068-13.2014

Demirtaş, M., Burt, J. B., Helmer, M., Ji, J. L., Adkinson, B. D., Glasser, M. F., Van Essen, D. C., Sotiropoulos, S. N., Anticevic, A., & Murray, J. D. (2019). Hierarchical Heterogeneity across Human Cortex Shapes Large-Scale Neural Dynamics. *Neuron*, *101*(6), 1181-1194.e13. https://doi.org/10.1016/j.neuron.2019.01.017

Fortin, J.-P., Parker, D., Tunç, B., Watanabe, T., Elliott, M. A., Ruparel, K., Roalf, D. R., Satterthwaite, T. D., Gur, R. C., Gur, R. E., Schultz, R. T., Verma, R., & Shinohara, R. T. (2017). Harmonization of multi-site diffusion tensor imaging data. *NeuroImage*, *161*, 149–170. https://doi.org/10.1016/j.neuroimage.2017.08.047

Hansen, N. (2006). The CMA Evolution Strategy: A Comparing Review. In J. A. Lozano, P. Larrañaga, I. Inza, & E. Bengoetxea (Eds.), *Towards a New Evolutionary Computation* (Vol. 192, pp. 75–102). Springer Berlin Heidelberg. https://doi.org/10.1007/3-540-32494-1_4

Heinzle, J., Koopmans, P. J., Den Ouden, H. E. M., Raman, S., & Stephan, K. E. (2016). A hemodynamic model for layered BOLD signals. *NeuroImage*, *125*, 556–570. https://doi.org/10.1016/j.neuroimage.2015.10.025

Kong, X., Kong, R., Orban, C., Wang, P., Zhang, S., Anderson, K., Holmes, A., Murray, J. D., Deco, G., Van Den Heuvel, M., & Yeo, B. T. T. (2021). Sensory-motor cortices shape functional connectivity dynamics in the human brain. *Nature Communications*, *12*(1), 6373. https://doi.org/10.1038/s41467-021-26704-y

Leonardi, N., & Van De Ville, D. (2015). On spurious and real fluctuations of dynamic functional connectivity during rest. *NeuroImage*, *104*, 430–436. https://doi.org/10.1016/j.neuroimage.2014.09.007

Liégeois, R., Laumann, T. O., Snyder, A. Z., Zhou, J., & Yeo, B. T. T. (2017). Interpreting temporal fluctuations in resting-state functional connectivity MRI. *NeuroImage*, *163*, 437–455. https://doi.org/10.1016/j.neuroimage.2017.09.012

Stephan, K. E., Weiskopf, N., Drysdale, P. M., Robinson, P. A., & Friston, K. J. (2007). Comparing hemodynamic models with DCM. *NeuroImage*, *38*(3), 387–401. https://doi.org/10.1016/j.neuroimage.2007.07.040

Zhang, S., Larsen, B., Sydnor, V. J., Zeng, T., An, L., Yan, X., Kong, R., Kong, X., Gur, R. C., Gur, R. E., Moore, T. M., Wolf, D. H., Holmes, A. J., Xie, Y., Zhou, J. H., Fortier, M. V., Tan, A. P., Gluckman, P., Chong, Y. S., … Yeo, B. T. T. (2024). In vivo whole-cortex marker of excitation-inhibition ratio indexes cortical maturation and cognitive ability in youth. *Proceedings of the National Academy of Sciences*, *121*(23), e2318641121. https://doi.org/10.1073/pnas.2318641121

## **Table S1. Participant characteristics in the ADNI dataset.** SD: standard deviation. FD: framewise displacement. CN: cognitively normal. MCI: mild cognitive impairment.

| **ADNI, N = 302** | |
| --- | --- |
| age, year (SD) | 72.7 (7.13) |
| sex (%) | male: 133 (44); female: 169 (56) |
| FD, mm (SD) | 0.12 (0.07) |
| education, year (SD) | 16.4 (2.54) |
| diagnosis (%) | CN: 194 (64); MCI: 108 (36) |
| CSF Aβ42, pg/mL (SD) | 1242.8 (664.8) |

## **Table S2. Imaging parameters of the 302 ADNI participants**

| Manufacturer | Model | TR (s) | Voxel size (mm) | Number of participants |
| --- | --- | --- | --- | --- |
| GE | Discovery MR750 | 3 | 3.4 | 57 |
|  | Discovery MR750w | 3 | 3.4 | 12 |
|  | Signa Premier | 3 | 3.4 | 7 |
| Philips | Achieva | 3 | 3.31-3.39 | 38 |
|  | Ingenia | 3 | 3.31-3.39 | 18 |
|  | Intera | 3 | 3.31 | 7 |
| Siemens | Prisma Fit | 0.607 | 2.5 | 45 |
|  | Prisma Fit | 3 | 3.4 | 58 |
|  | Prisma | 0.607 | 2.5 | 22 |
|  | Prisma | 3 | 3.4 | 26 |
|  | Verio | 3 | 3.4 | 12 |

## **Table S3. Participant characteristics in the MACC dataset.** SD: standard deviation. FD: framewise displacement. CN: cognitively normal. MCI: mild cognitive impairment.

| **MACC, N = 240** | |
| --- | --- |
| age, year (SD) | 71.9 (7.56) |
| sex (%) | male: 107 (45); female: 133 (55) |
| FD, mm (SD) | 0.069 (0.046) |
| education, year (SD) | 8.93 (4.95) |
| diagnosis (%) | CN: 95 (40); MCI: 145 (60) |
| plasma p-tau 217, pg/mL (SD) | 0.494 (0.388) |

## **Table S4. Cognitive measures included in the MACC memory score and their factor loadings.** Each measure was z-scored across participants, and a single latent factor was estimated by maximum-likelihood factor analysis. Measures are listed in descending order of factor loading. The latent factor accounted for 54.4% of the variance.

| Word List Recall | Immediate recall |
| --- | --- |
|  | Delayed recall |
| Story Recall | Immediate recall |
|  | Delayed recall |
| MMSE Orientation | Day of the week Today |
|  | Date Today |
|  | Current Month |
|  | Current Year |
|  | Place (Clinic/Hospital) |
|  | Time |
|  | Name of the place |
|  | Estate |
|  | Floor |
|  | Country |
| MMSE Registration & Recall | 3-object registration (Lemon, Key, Balloon) |
|  | Recall Score |

|  | **Factor loading** |
| --- | --- |
| Story Recall | 0.798 |
| Word List Recall | 0.782 |
| MMSE Registration & Recall | 0.688 |
| MMSE Orientation | 0.674 |

## **Table S5. Association between mean cortical E/I ratio and CSF Aβ42.** Unlike Figure 2(A), the E/I ratios here were not harmonized across sites. General linear model (GLM) between mean cortical E/I ratio and log-transformed CSF Aβ42, adjusting for age, sex (female = 0, male = 1), head motion (i.e., FD) and years of education. An intercept term is included. All continuous variables are z-scored across all participants prior to GLM fitting. SE: standard errors.

|  | **regression coefficient (β)** | **SE** | **t** | ***p*** |
| --- | --- | --- | --- | --- |
| intercept | 0.0637 | 0.0758 | 0.840 | 0.401 |
| log_10_(CSF Aβ_42_) | -0.181 | 0.0569 | -3.17 | 0.00168 |
| age | 0.123 | 0.0574 | 2.15 | 0.0325 |
| sex | -0.145 | 0.115 | -1.25 | 0.211 |
| FD | 0.0560 | 0.0570 | 0.983 | 0.326 |
| education | -0.0336 | 0.0567 | -0.592 | 0.554 |

## **Table S6. Association between mean cortical E/I ratio and CSF Aβ42.** General linear model (GLM) between mean cortical E/I ratio and log-transformed CSF Aβ42, adjusting for age, sex (female = 0, male = 1), head motion (i.e., FD), years of education, BMI, hypertension (no = 0, yes = 1), diabetes (no = 0, yes = 1) and smoking history (no = 0, yes = 1). An intercept term is included. All continuous variables are z-scored across all participants prior to GLM fitting. SE: standard errors.

|  | **regression coefficient (β)** | **SE** | **t** | ***p*** |
| --- | --- | --- | --- | --- |
| intercept | 0.335 | 0.141 | 2.38 | 0.0181 |
| log_10_(CSF Aβ_42_) | -0.184 | 0.0571 | -3.22 | 0.00141 |
| age | 0.146 | 0.0604 | 2.42 | 0.0161 |
| sex | -0.210 | 0.118 | -1.79 | 0.0752 |
| FD | -0.00779 | 0.0602 | -0.129 | 0.897 |
| education | -0.000190 | 0.0574 | -0.00331 | 0.997 |
| BMI | 0.0998 | 0.0618 | 1.62 | 0.107 |
| hypertension | -0.189 | 0.126 | -1.51 | 0.133 |
| diabetes | -0.242 | 0.142 | -1.70 | 0.0896 |
| smoking history | -0.0873 | 0.197 | -0.444 | 0.657 |

## **Table S7. Association between mean cortical E/I ratio and CSF Aβ42/40 ratio.** General linear model (GLM) between mean cortical E/I ratio and log-transformed CSF Aβ42/40 ratio, adjusting for age, sex (female = 0, male = 1), head motion (i.e., FD) and years of education. An intercept term is included. All continuous variables are z-scored across all participants prior to GLM fitting. SE: standard errors.

|  | **regression coefficient (β)** | **SE** | **t** | ***p*** |
| --- | --- | --- | --- | --- |
| intercept | 0.0935 | 0.0819 | 1.14 | 0.255 |
| log_10_(CSF Aβ_42_:Aβ_40_) | -0.134 | 0.0632 | -2.12 | 0.0347 |
| age | 0.146 | 0.0643 | 2.26 | 0.0244 |
| sex | -0.216 | 0.125 | -1.72 | 0.0863 |
| FD | -0.0348 | 0.0620 | -0.562 | 0.574 |
| education | -0.0319 | 0.0619 | -0.515 | 0.607 |

## **Table S8. Association between mean cortical excitation (*S_E_*) and CSF Aβ42.** General linear model (GLM) between mean cortical excitation and log-transformed CSF Aβ42, adjusting for age, sex (female = 0, male = 1), head motion (i.e., FD) and years of education. An intercept term is included. All continuous variables are z-scored across all participants prior to GLM fitting. SE: standard errors.

|  | **regression coefficient (β)** | **SE** | **t** | ***p*** |
| --- | --- | --- | --- | --- |
| intercept | 0.00322 | 0.0774 | -0.0417 | 0.967 |
| log_10_(CSF Aβ_42_) | 0.0185 | 0.0581 | 0.319 | 0.750 |
| age | 0.0877 | 0.0586 | 1.50 | 0.135 |
| sex | 0.00732 | 0.118 | 0.0623 | 0.950 |
| FD | 0.0969 | 0.0581 | 1.67 | 0.0965 |
| education | 0.0240 | 0.0579 | 0.415 | 0.678 |

## **Table S9. Association between mean cortical inhibition (*S_I_*) and CSF Aβ42.** General linear model (GLM) between mean cortical inhibition and log-transformed CSF Aβ42, adjusting for age, sex (female = 0, male = 1), head motion (i.e., FD) and years of education. An intercept term is included. All continuous variables are z-scored across all participants prior to GLM fitting. SE: standard errors.

|  | **regression coefficient (β)** | **SE** | **t** | ***p*** |
| --- | --- | --- | --- | --- |
| intercept | -0.0988 | 0.0757 | -1.30 | 0.193 |
| log_10_(CSF Aβ_42_) | 0.173 | 0.0569 | 3.04 | 0.00255 |
| age | -0.147 | 0.0573 | -2.56 | 0.0109 |
| sex | 0.224 | 0.115 | 1.95 | 0.0523 |
| FD | 0.0174 | 0.0569 | 0.305 | 0.760 |
| education | 0.00773 | 0.0567 | 0.135 | 0.892 |

## **Figure S10.** (A) Parvalbumin (PVALB) gene expression map (Anderson et al., 2020). (B) Agreement between the magnitude of E/I-ratio-CSF-Aβ42 slope and PVALB expression. *r* = 0.359, two-tailed spin test *p* = 0.202.

## **Figure S11. Spatial distribution of the associations between E/I ratio and CSF Aβ42.** Unlike Figure 3, the E/I ratios here were not harmonized across sites. (A) General linear model between regional cortical E/I ratio and log-transformed CSF Aβ42, adjusting for age, sex (female = 0, male = 1), head motion (i.e., FD) and years of education. All regression coefficients (slopes) were negative and statistically significant (FDR *q* < 0.05). (B) The slopes exhibited a spatial structure with lower-order sensory networks showing the largest (most negative) slopes. The boxes show the interquartile range (IQR) and the median. Whiskers indicate 1.5 IQR. Black crosses represent outliers. (C) Agreement between the E/I-ratio-CSF-Aβ42 slope (panel A) and SA axis rank. Spearman’s correlation *r* = 0.863, two-tailed spin test *p* < 0.001.

## **Figure S12. Spatial distribution of the associations between E/I ratio and CSF Aβ42.** (A) General linear model between regional cortical E/I ratio and log-transformed CSF Aβ42, adjusting for age, sex (female = 0, male = 1), head motion (i.e., FD), years of education, BMI, hypertension (no = 0, yes = 1), diabetes (no = 0, yes = 1) and smoking history (no = 0, yes = 1). All regression coefficients (slopes) were negative and statistically significant (FDR *q* < 0.05). (B) The slopes exhibited a spatial structure with lower-order sensory networks showing the largest (most negative) slopes. The boxes show the interquartile range (IQR) and the median. Whiskers indicate 1.5 IQR. Black crosses represent outliers. (C) Agreement between the E/I-ratio-CSF-Aβ42 slope (panel A) and SA axis rank. Spearman’s correlation *r* = 0.860, two-tailed spin test *p* < 0.001.

## **Figure S13. Spatial distribution of the associations between E/I ratio and CSF Aβ42/40 ratio.** (A) General linear model between regional cortical E/I ratio and log-transformed CSF Aβ42/40 ratio, adjusting for age, sex (female = 0, male = 1), head motion (i.e., FD) and years of education. All regression coefficients (slopes) were negative but not statistically significant (FDR *q* < 0.05). (B) The slopes exhibited a spatial structure with lower-order sensory networks showing the largest (most negative) slopes. The boxes show the interquartile range (IQR) and the median. Whiskers indicate 1.5 IQR. Black crosses represent outliers. (C) Agreement between the E/I-ratio-CSF-Aβ42/40 slope (panel A) and SA axis rank. Spearman’s correlation *r* = 0.877, two-tailed spin test *p* < 0.001.

## **Figure S14. Mediation analyses linking CSF Aβ42, E/I ratio, and cognition.** Unlike Figure 4, the E/I ratios here were not harmonized across sites. (A) Schematic of the mediation model testing whether mean cortical E/I ratio mediates the association between log-transformed CSF Aβ42 and cognitive performance (PHC memory score). Standardized regression coefficients are shown for path *a* (CSF Aβ42 → E/I ratio), path *b* (E/I ratio → cognition), and the direct effect *c′* (CSF Aβ42 → cognition, controlling for E/I ratio). All paths were adjusted for age, sex, head motion and years of education. All continuous variables were z-scored across participants. *p*-values were obtained from 10,000 bootstrap samples, * indicates *p* < 0.05; ** indicates *p* < 0.01; *** indicates *p* < 0.001. The proportion mediated was 21.9%. (B) Regional standardized coefficients for path *b* (regional E/I ratio → cognition). (C) Regional indirect effects (*a × b*). Regions that did not survive false discovery rate (FDR) correction across 68 cortical regions (*q* < 0.05) are shown in gray.

## **Figure S15. Mediation analyses linking CSF Aβ42, E/I ratio, and cognition.** (A) Schematic of the mediation model testing whether mean cortical E/I ratio mediates the association between log-transformed CSF Aβ42 and cognitive performance (PHC memory score). Standardized regression coefficients are shown for path *a* (CSF Aβ42 → E/I ratio), path *b* (E/I ratio → cognition), and the direct effect *c′* (CSF Aβ42 → cognition, controlling for E/I ratio). All paths were adjusted for age, sex, head motion**,** years of education, BMI, hypertension, diabetes and smoking history. All continuous variables were z-scored across participants**.** *p*-values were obtained from 10,000 bootstrap samples, * indicates *p* < 0.05; ** indicates *p* < 0.01; *** indicates *p* < 0.001. The proportion mediated was 14.7%. (B) Regional standardized coefficients for path *b* (regional E/I ratio → cognition). (C) Regional indirect effects (*a × b*). All regions survive false discovery rate (FDR) correction across 68 cortical regions (*q* < 0.05).

## **Figure S16. Mediation analyses linking CSF Aβ42/40 ratio, E/I ratio, and cognition.** (A) Schematic of the mediation model testing whether mean cortical E/I ratio mediates the association between log-transformed CSF Aβ42/40 and cognitive performance (PHC memory score). Standardized regression coefficients are shown for path *a* (CSF Aβ42/40 → E/I ratio), path *b* (E/I ratio → cognition), and the direct effect *c′* (CSF Aβ42/40 → cognition, controlling for E/I ratio). All paths were adjusted for age, sex, head motion and years of education. All continuous variables were z-scored across participants. *p*-values were obtained from 10,000 bootstrap samples, * indicates *p* < 0.05; ** indicates *p* < 0.01; *** indicates *p* < 0.001. The proportion mediated was 18.9%. (B) Regional standardized coefficients for path *b* (regional E/I ratio → cognition). (C) Regional indirect effects (*a × b*). All regional coefficients of path *b* survived FDR correction across 68 regions (*q* < 0.05), but none of the regional coefficients of path *a* *× b* survived FDR correction across 68 regions (*q* < 0.05).

## **Table S17. Association between mean cortical E/I ratio and plasma p-tau217.** General linear model (GLM) between mean cortical E/I ratio and log-transformed plasma p-tau217, adjusting for age, sex (female = 0, male = 1), head motion (i.e., FD), years of education, BMI, hypertension (no = 0, yes = 1), diabetes (no = 0, yes = 1) and smoking history (no = 0, yes = 1). An intercept term is included. All continuous variables are z-scored across all participants prior to GLM fitting. SE: standard errors.

|  | **regression coefficient (β)** | **SE** | **t** | ***p*** |
| --- | --- | --- | --- | --- |
| intercept | 0.0795 | 0.116 | 0.688 | 0.492 |
| log_10_(plasma p-tau_217_) | 0.187 | 0.0730 | 2.56 | 0.0110 |
| age | -0.0717 | 0.0759 | -0.944 | 0.346 |
| sex | 0.0418 | 0.161 | 0.260 | 0.795 |
| FD | -0.191 | 0.0663 | -2.89 | 0.00427 |
| education | -0.101 | 0.0667 | -1.51 | 0.131 |
| BMI | 0.0357 | 0.0646 | 0.553 | 0.581 |
| hypertension | -0.170 | 0.136 | -1.25 | 0.213 |
| diabetes | 0.137 | 0.145 | 0.940 | 0.348 |
| smoking history | -0.161 | 0.190 | -0.846 | 0.400 |

## **Table S18. Association between mean cortical E/I ratio and plasma p-tau181.** General linear model (GLM) between mean cortical E/I ratio and log-transformed plasma p-tau181, adjusting for age, sex (female = 0, male = 1), head motion (i.e., FD) and years of education. An intercept term is included. All continuous variables are z-scored across all participants prior to GLM fitting. SE: standard errors.

|  | **regression coefficient (β)** | **SE** | **t** | ***p*** |
| --- | --- | --- | --- | --- |
| intercept | 0.0358 | 0.0852 | 0.420 | 0.675 |
| log_10_(plasma p-tau_181_) | 0.143 | 0.0693 | 2.06 | 0.0404 |
| age | -0.0351 | 0.0690 | -0.508 | 0.612 |
| sex | -0.0800 | 0.130 | -0.616 | 0.538 |
| FD | -0.189 | 0.0643 | -2.94 | 0.00364 |
| education | -0.101 | 0.0648 | -1.56 | 0.119 |

## **Table S19. Association between mean cortical excitation (*S_E_*) and plasma p-tau217.** General linear model (GLM) between mean cortical E/I ratio and log-transformed plasma p-tau217, adjusting for age, sex (female = 0, male = 1), head motion (i.e., FD) and years of education. An intercept term is included. All continuous variables are z-scored across all participants prior to GLM fitting. SE: standard errors.

|  | **regression coefficient (β)** | **SE** | **t** | ***p*** |
| --- | --- | --- | --- | --- |
| intercept | 0.0350 | 0.0885 | 0.396 | 0.692 |
| log_10_(plasma p-tau_217_) | 0.0329 | 0.0724 | 0.454 | 0.650 |
| age | -0.0628 | 0.0733 | -0.856 | 0.393 |
| sex | -0.0786 | 0.135 | -0.581 | 0.562 |
| FD | -0.0950 | 0.0661 | -1.44 | 0.152 |
| education | -0.00104 | 0.0673 | -0.0155 | 0.988 |

## **Table S20. Association between mean cortical inhibition (*S_I_*) and plasma p-tau217.** General linear model (GLM) between mean cortical E/I ratio and log-transformed plasma p-tau217, adjusting for age, sex (female = 0, male = 1), head motion (i.e., FD) and years of education. An intercept term is included. All continuous variables are z-scored across all participants prior to GLM fitting. SE: standard errors.

|  | **regression coefficient (β)** | **SE** | **t** | ***p*** |
| --- | --- | --- | --- | --- |
| intercept | -0.0115 | 0.0860 | -0.134 | 0.894 |
| log_10_(plasma p-tau_217_) | -0.158 | 0.0704 | -2.24 | 0.0262 |
| age | 0.0455 | 0.0713 | 0.638 | 0.524 |
| sex | 0.0258 | 0.131 | 0.196 | 0.845 |
| FD | 0.209 | 0.0642 | 3.26 | 0.00130 |
| education | 0.108 | 0.0654 | 1.66 | 0.0988 |

## **Figure S21.** (A) Parvalbumin (PVALB) gene expression map (Anderson et al., 2020). (B) Agreement between the magnitude of E/I-ratio-plasma-ptau-217 slope and PVALB expression. *r* = 0.403, two-tailed spin test *p* = 0.116.

## **Figure S22. Spatial distribution of the associations between E/I ratio and plasma p-tau217.** (A) General linear model between regional cortical E/I ratio and log-transformed plasma p-tau217, adjusting for age, sex (female = 0, male = 1), head motion (i.e., FD), years of education, BMI, hypertension (no = 0, yes = 1), diabetes (no = 0, yes = 1) and smoking history (no = 0, yes = 1). All regression coefficients (slopes) were positive and statistically significant (FDR *q* < 0.05). (B) The slopes exhibited a spatial structure with lower-order sensory networks showing the largest (most positive) slopes. The boxes show the interquartile range (IQR) and the median. Whiskers indicate 1.5 IQR. Black crosses represent outliers. (C) Agreement between the E/I-ratio-plasma-ptau217 slope (panel A) and SA axis rank. Spearman’s correlation *r* = -0.791, two-tailed spin test *p* = 0.002.

## **Figure S23. Spatial distribution of the associations between E/I ratio and plasma p-tau181.** (A) General linear model between regional cortical E/I ratio and log-transformed plasma p-tau217, adjusting for age, sex (female = 0, male = 1), head motion (i.e., FD) and years of education. All regression coefficients (slopes) were positive and but not statistically significant (FDR *q* < 0.05). (B) The slopes exhibited a spatial structure with lower-order sensory networks showing the largest (most positive) slopes. The boxes show the interquartile range (IQR) and the median. Whiskers indicate 1.5 IQR. Black crosses represent outliers. (C) Agreement between the E/I-ratio-plasma-ptau181 slope (panel A) and SA axis rank. Spearman’s correlation *r* = -0.893, two-tailed spin test *p* < 0.001.

## **Figure S24. Mediation analyses linking plasma p-tau217, E/I ratio, and cognition.** (A) Schematic of the mediation model testing whether mean cortical E/I ratio mediates the association between log-transformed p-tau217 and cognitive performance (memory score). Standardized regression coefficients are shown for path *a* (plasma p-tau217 → E/I ratio), path *b* (E/I ratio → cognition), and the direct effect *c′* (plasma p-tau217→ cognition, controlling for E/I ratio). All paths were adjusted for age, sex, head motion, years of education, BMI, hypertension, diabetes and smoking history. All continuous variables were z-scored across participants. *p*-values were obtained from 10,000 bootstrap samples, * indicates *p* < 0.05; ** indicates *p* < 0.01; *** indicates *p* < 0.001. The proportion mediated was 5.26%. (B) Regional standardized coefficients for path *b* (regional E/I ratio → cognition). Regions that did not survive false discovery rate (FDR) correction across 68 cortical regions (*q* < 0.05) are shown in gray. (C) Regional indirect effects (*a × b*). None of the regional coefficients of path *a* *× b* survived FDR correction across 68 regions (*q* < 0.05)

## **Figure S25. Mediation analyses linking plasma p-tau181, E/I ratio, and cognition.** (A) Schematic of the mediation model testing whether mean cortical E/I ratio mediates the association between log-transformed plasma p-tau181 and cognitive performance (memory score). Standardized regression coefficients are shown for path *a* (plasma p-tau181 → E/I ratio), path *b* (E/I ratio → cognition), and the direct effect *c′* (plasma p-tau181 → cognition, controlling for E/I ratio). All paths were adjusted for age, sex, head motion and years of education. All continuous variables were z-scored across participants. *p*-values were obtained from 10,000 bootstrap samples, * indicates *p* < 0.05; ** indicates *p* < 0.01; *** indicates *p* < 0.001; ns indicates *p* > 0.05. The proportion mediated was 8.06%. (B) Regional standardized coefficients for path *b* (regional E/I ratio → cognition). (C) Regional indirect effects (*a × b*). All regional coefficients of path *b* and path *a* *× b* survived FDR correction across 68 regions (*q* < 0.05).

## **Table S26. Global mediation analyses using each constituent measure of the MACC memory composite and its corresponding ADNI measure.** The mediation relating the amyloid marker to cognition through the mean cortical E/I ratio was repeated using each individual measure that makes up the memory composite, in each cohort. The independent variable was the log-transformed CSF Aβ42 in ADNI and plasma p-tau217 in MACC, the mediator was the mean cortical E/I ratio, and the dependent variable was the cognitive measure indicated in each row. The first row of each cohort reproduces the composite-score analysis for reference (ADNI: ADSP-PHC memory score; MACC: the single-factor memory composite). Each MACC constituent measure is paired with its closest ADNI counterpart: Story Recall with Logical Memory, Word List Recall with RAVLT, and the shared MMSE Registration & Recall and MMSE Orientation. For each analysis, standardized coefficients (β) and bootstrap *p*-values are given for path *a* (biomarker → E/I ratio), path *b* (E/I ratio → cognition), the direct effect *c′* (biomarker → cognition, adjusting for E/I ratio), and the indirect effect *a × b*; the proportion mediated is the indirect effect as a fraction of the total effect. All models adjusted for age, sex, head motion and years of education, and all continuous variables were z-scored across participants. *p*-values were obtained from 10,000 bootstrap samples. Sample sizes differ across the ADNI measures because of differing availability of each test; all MACC analyses used the same 240 participants. Bold font indicates significant *p*-values after FDR correction (*q* < 0.05) across all path coefficients in the table.

| **Cohort** | **Cognitive measure** | **N** | **path  *a*, β  (*p*)** | **path *b*, β  (*p*)** | **path  *c’*, β  (*p*)** | **indirect effect *a × b*, β  (*p*)** | **Proportion mediated** |
| --- | --- | --- | --- | --- | --- | --- | --- |
| ADNI | ADSP-PHC  Memory | 190 | **-0.228 (0.0008)** | **-0.141 (0.0248)** | **0.188 (0.0080)** | **0.032 (0.0256)** | 14.5% |
| ADNI | Logical Memory | 255 | **-0.180**  **(0.0042)** | -0.066 (0.2534) | **0.262 (<1e-4)** | 0.012 (0.2568) | 4.31% |
| ADNI | RAVLT | 180 | **-0.242 (0.0010)** | -0.114 (0.0880) | 0.114 (0.1112) | 0.028 (0.089) | 19.6% |
| ADNI | MMSE Registration & Recall | 302 | **-0.177 (0.0028)** | -0.042 (0.4174) | 0.015 (0.8124) | 0.007 (0.4190) | 32.6% |
| ADNI | MMSE Orientation | 302 | **-0.177 (0.0028)** | -0.114 (0.0372) | **0.163 (0.0054)** | 0.020 (0.0400) | 11.0% |
| MACC | Composite Memory | 240 | **0.176 (0.0120)** | **-0.120 (0.0172)** | **-0.391 (<1e-4)** | **-0.021 (0.0292)** | 5.11% |
| MACC | Story  Recall | 240 | **0.176 (0.0120)** | **-0.115 (0.0156)** | **-0.277 (<1e-4)** | **-0.020 (0.0276)** | 6.82% |
| MACC | Word List Recall | 240 | **0.176 (0.0120)** | -0.110 (0.0392) | **-0.288 (<1e-4)** | -0.019 (0.0512) | 6.30% |
| MACC | MMSE Registration & Recall | 240 | **0.176 (0.0120)** | -0.116 (0.0464) | **-0.381 (<1e-4)** | -0.020 (0.0580) | 5.08% |
| MACC | MMSE Orientation | 240 | **0.176 (0.0120)** | -0.030 (0.6622) | **-0.387 (<1e-4)** | -0.005 (0.6630) | 1.33% |

## **Table S27. Cognitive measures included in the MACC-matched ADNI composite memory score.**

| Logical Memory Test | Immediate recall |
| --- | --- |
|  | Delayed recall |
| RAVLT | Immediate recall |
|  | Delayed recall |
| MMSE Orientation | Day of the week Today |
|  | Date Today |
|  | Current Month |
|  | Current Season |
|  | Place (Clinic/Hospital) |
|  | Time |
|  | City |
|  | State |
|  | Floor |
|  | Country |
| MMSE Registration & Recall | 3-object registration (Ball, Flag, Tree) |
|  | Recall Score |

## **Figure S28. Mediation analyses linking CSF Aβ42, E/I ratio, and cognition, using the MACC-matched ADNI composite memory score.** (A) Schematic of the mediation model testing whether mean cortical E/I ratio mediates the association between log-transformed CSF Aβ42 and cognitive performance (composite memory score). Standardized regression coefficients are shown for path *a* (CSF Aβ42 → E/I ratio), path *b* (E/I ratio → cognition), and the direct effect *c′* (CSF Aβ42 → cognition, controlling for E/I ratio). All paths were adjusted for age, sex, head motion and years of education. All continuous variables were z-scored across participants. *p*-values were obtained from 10,000 bootstrap samples; ** indicates *p* < 0.01 and n.s. indicates *p* ≥ 0.05. The indirect effect (*a × b*) did not reach significance. The proportion mediated (ratio of indirect to total effects) was 0.0288/(0.0288+0.204) = 12.4%. (B) Regional standardized coefficients for path *b* (regional E/I ratio → cognition). (C) Regional indirect effects (*a × b*). None of the regional coefficients for path *b* or (*a × b*) survived FDR correction across the 68 regions (*q* < 0.05).
